# Supplementary figures and images for: Steroidal glycoalkaloids from Solanum nigrum target cytoskeletal proteins: an in silico analysis
Source: PeerJ. 2019 Jan 3;7:e6012. doi: 10.7717/peerj.6012 (PMC6321755; doi:10.7717/peerj.6012)

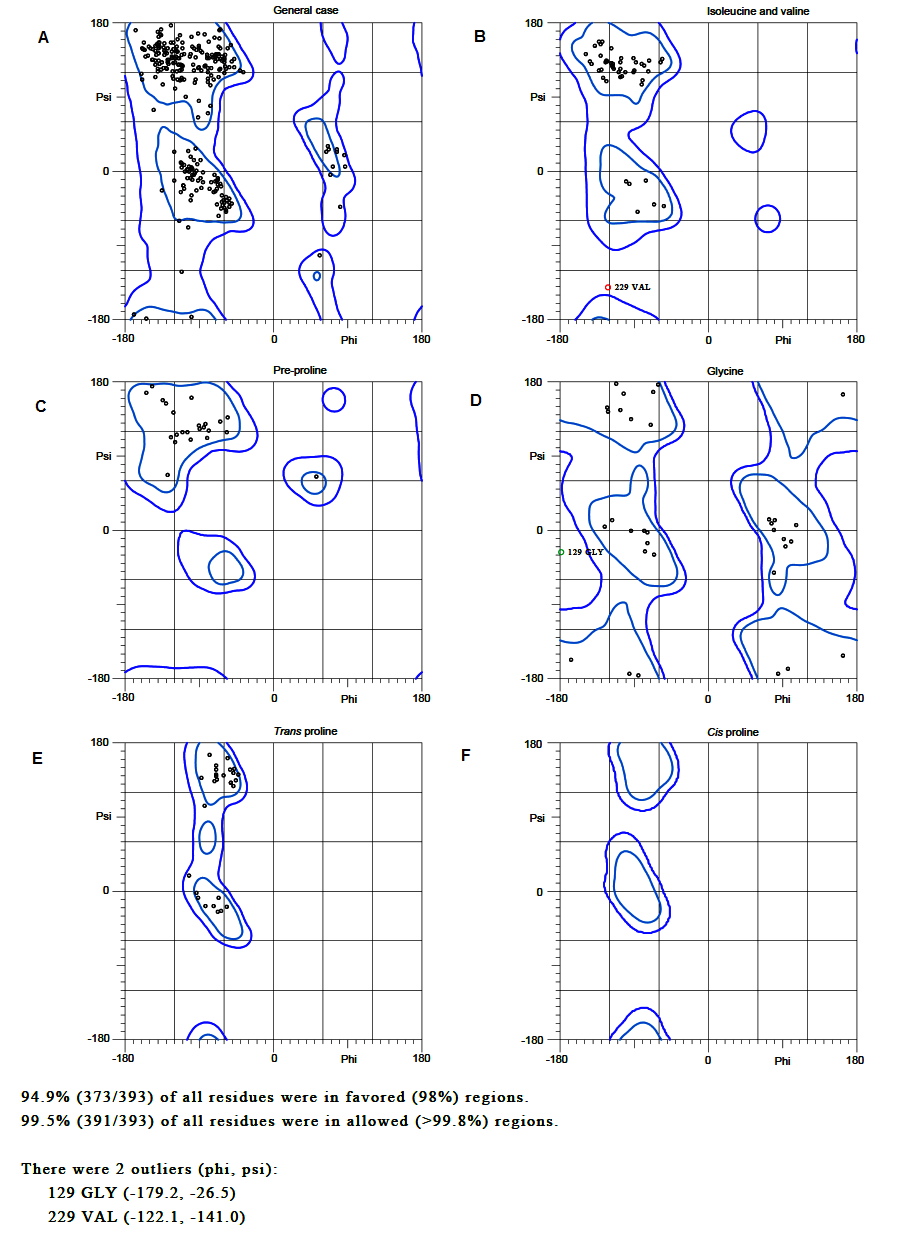

Supplement: Figure S1 [file peerj-07-6012-s001.png]

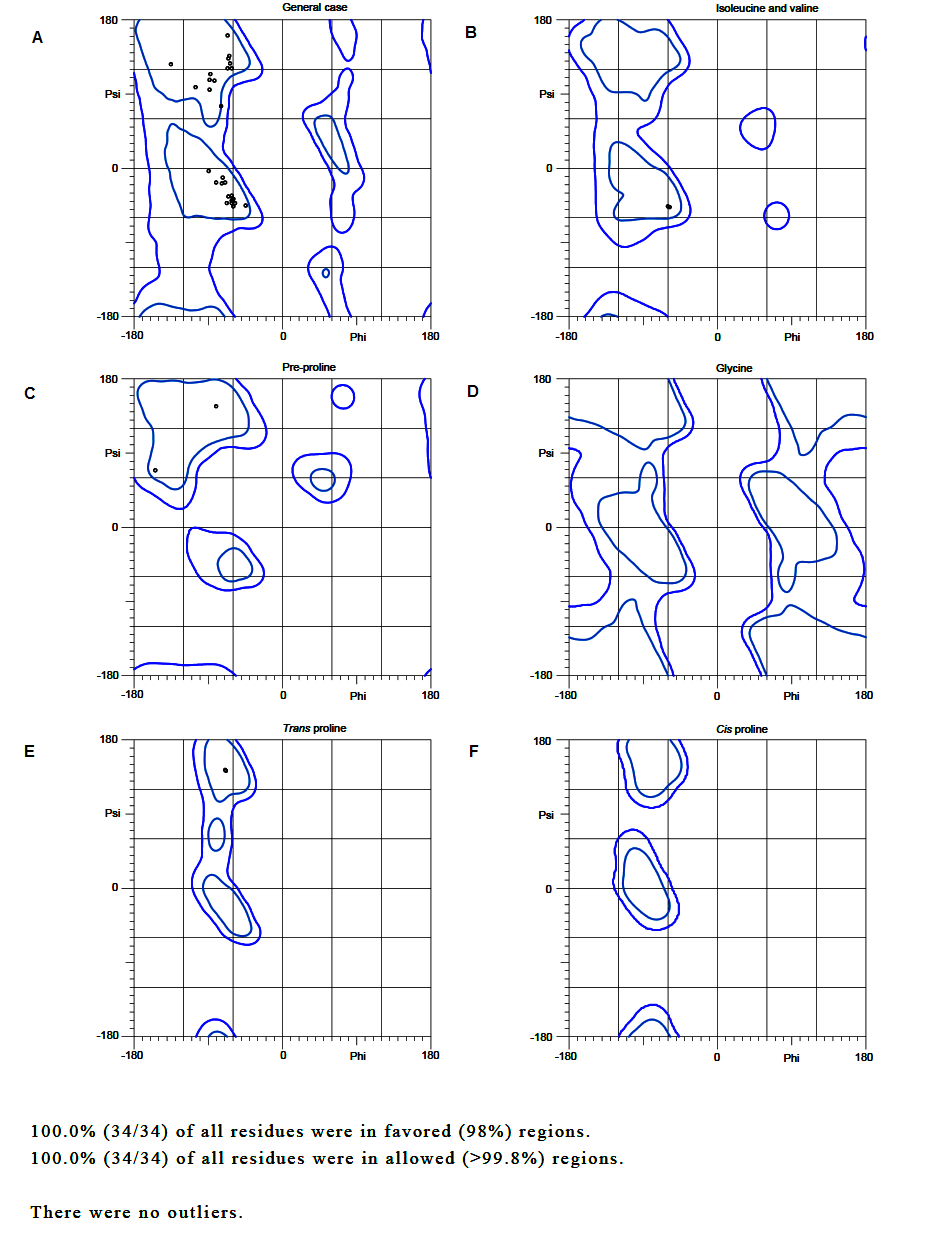

Supplement: Figure S2 [file peerj-07-6012-s002.png]

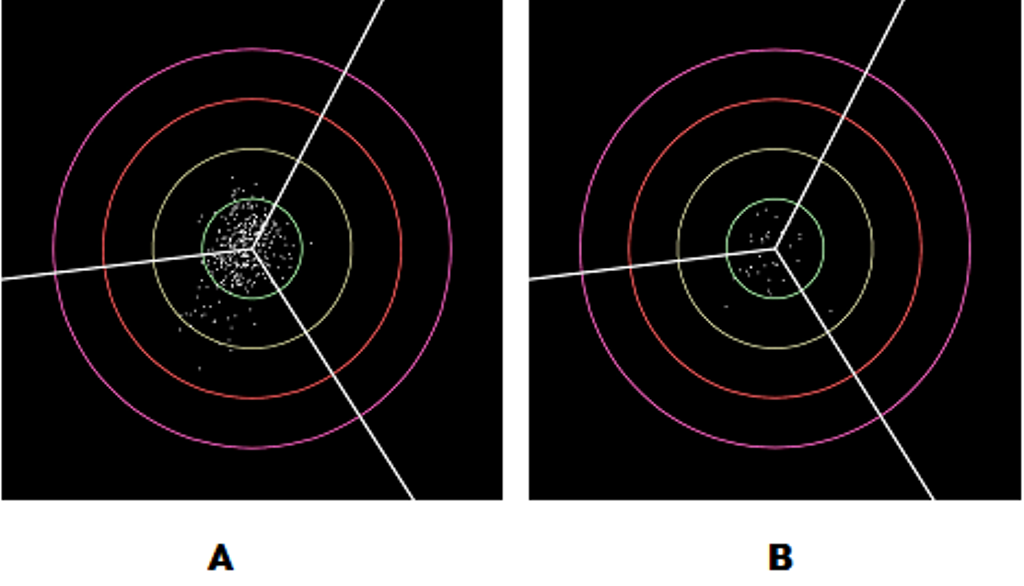

Supplement: Figure S3 [file peerj-07-6012-s003.png]

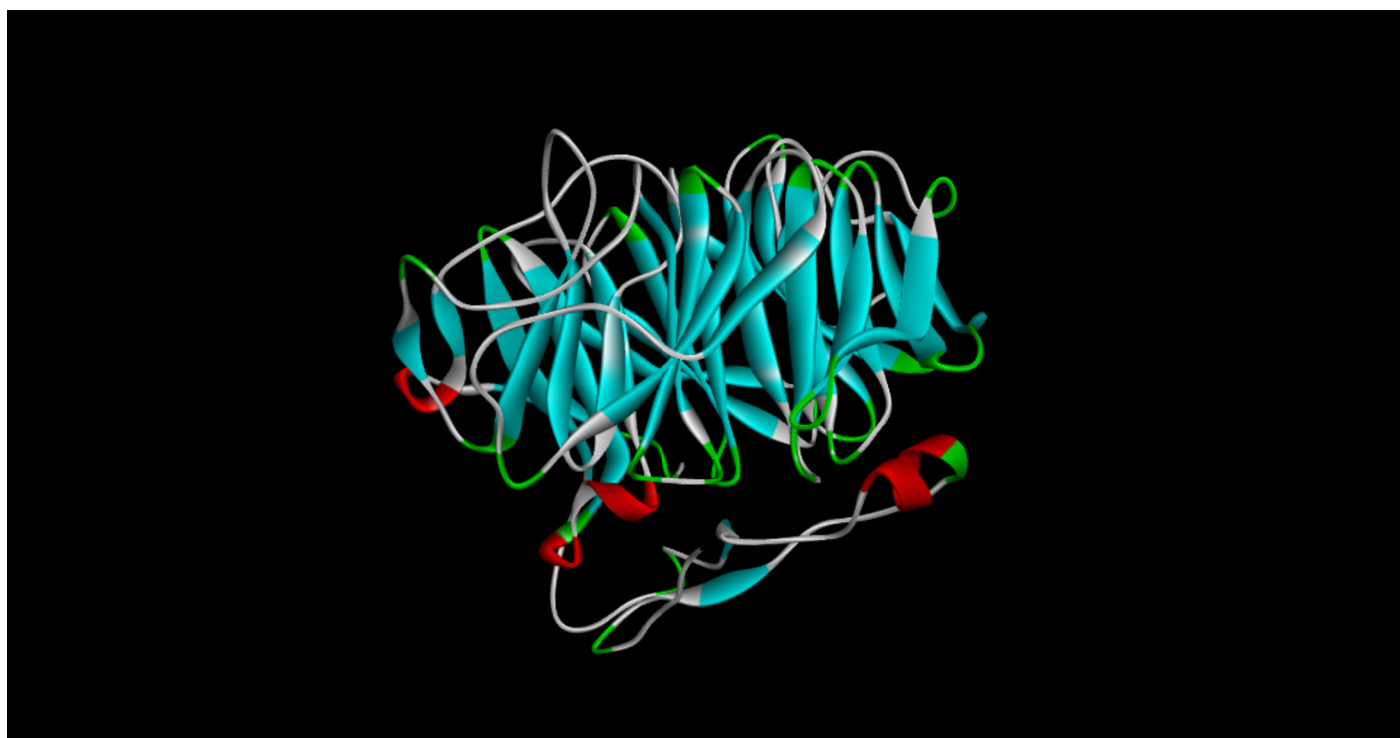

Supplement: Figure S12 [file peerj-07-6012-s012.pdf]

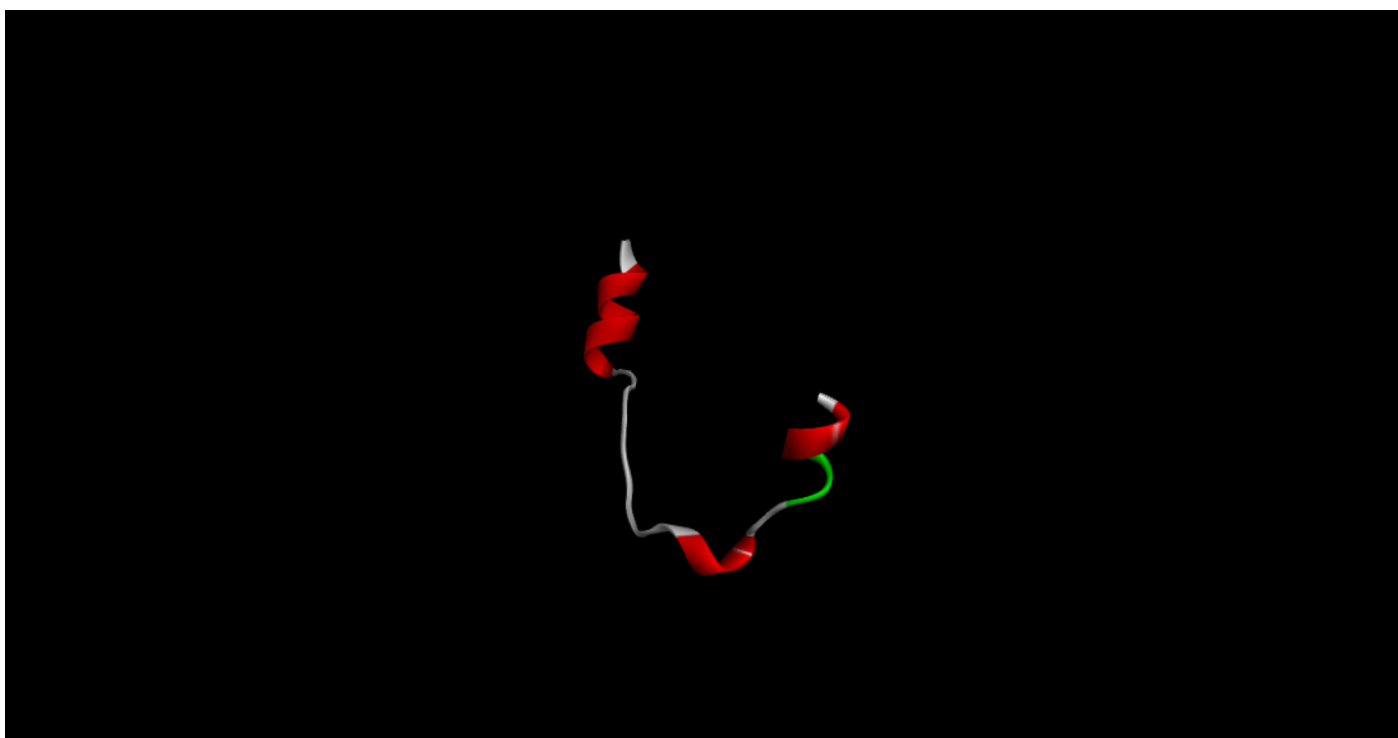

Supplement: Figure S13 [file peerj-07-6012-s013.pdf]

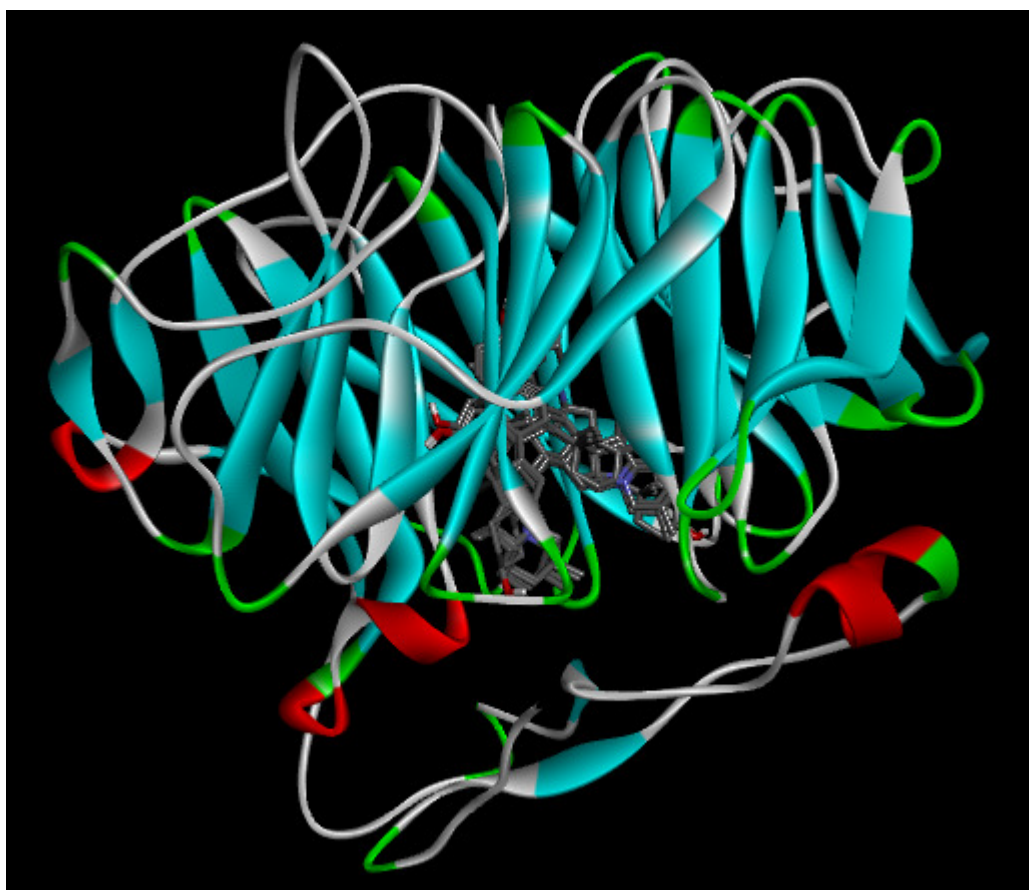

Supplement: Figure S14 [file peerj-07-6012-s014.pdf]

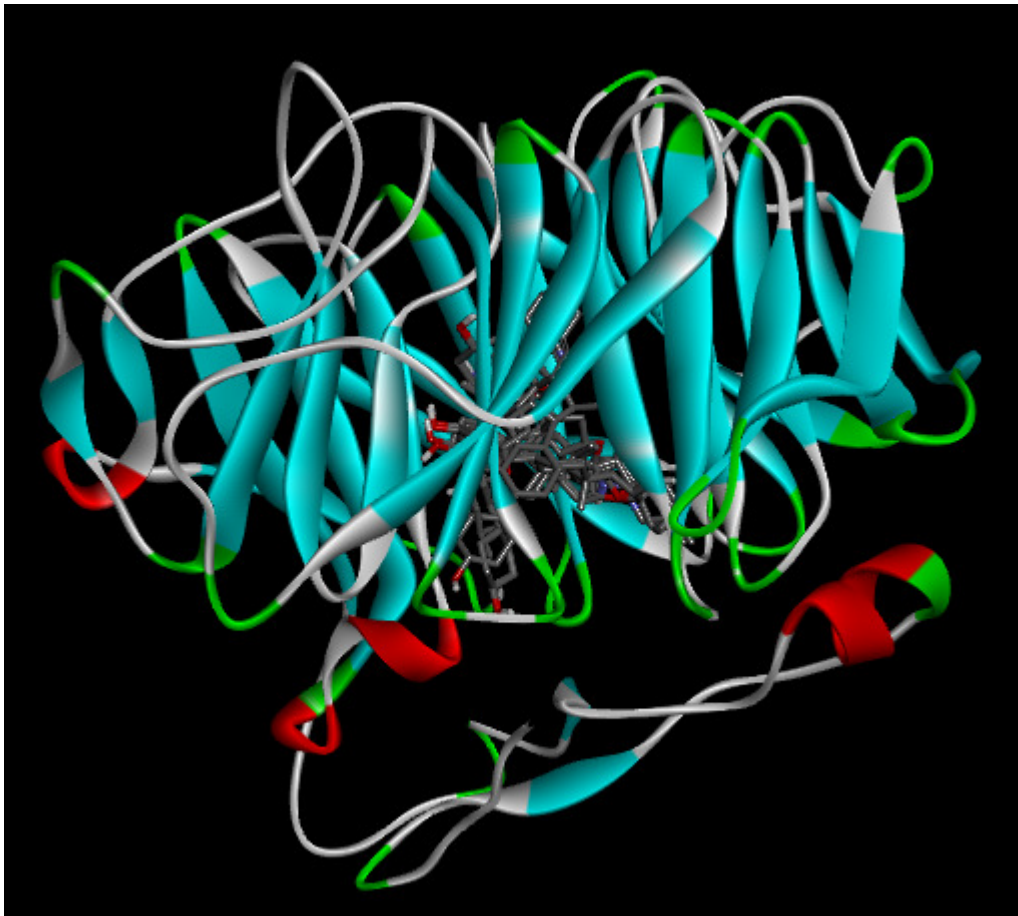

Supplement: Figure S15 [file peerj-07-6012-s015.pdf]

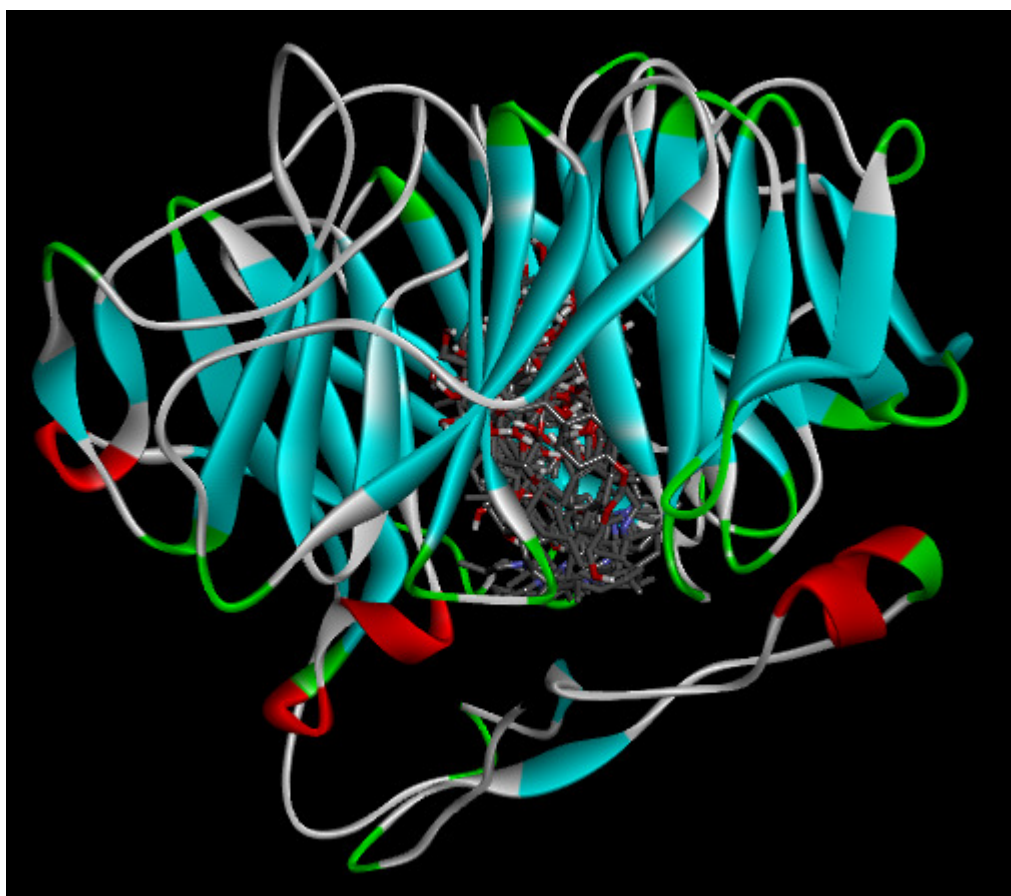

Supplement: Figure S16 [file peerj-07-6012-s016.pdf]

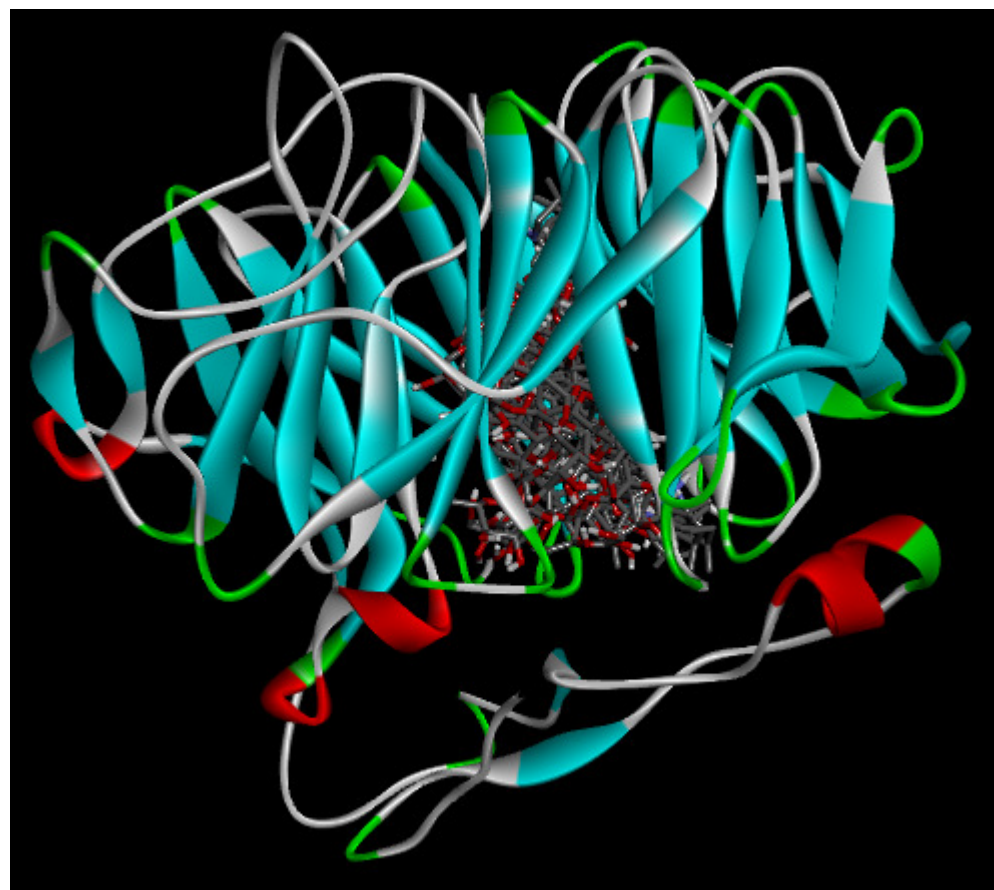

Supplement: Figure S17 [file peerj-07-6012-s017.pdf]

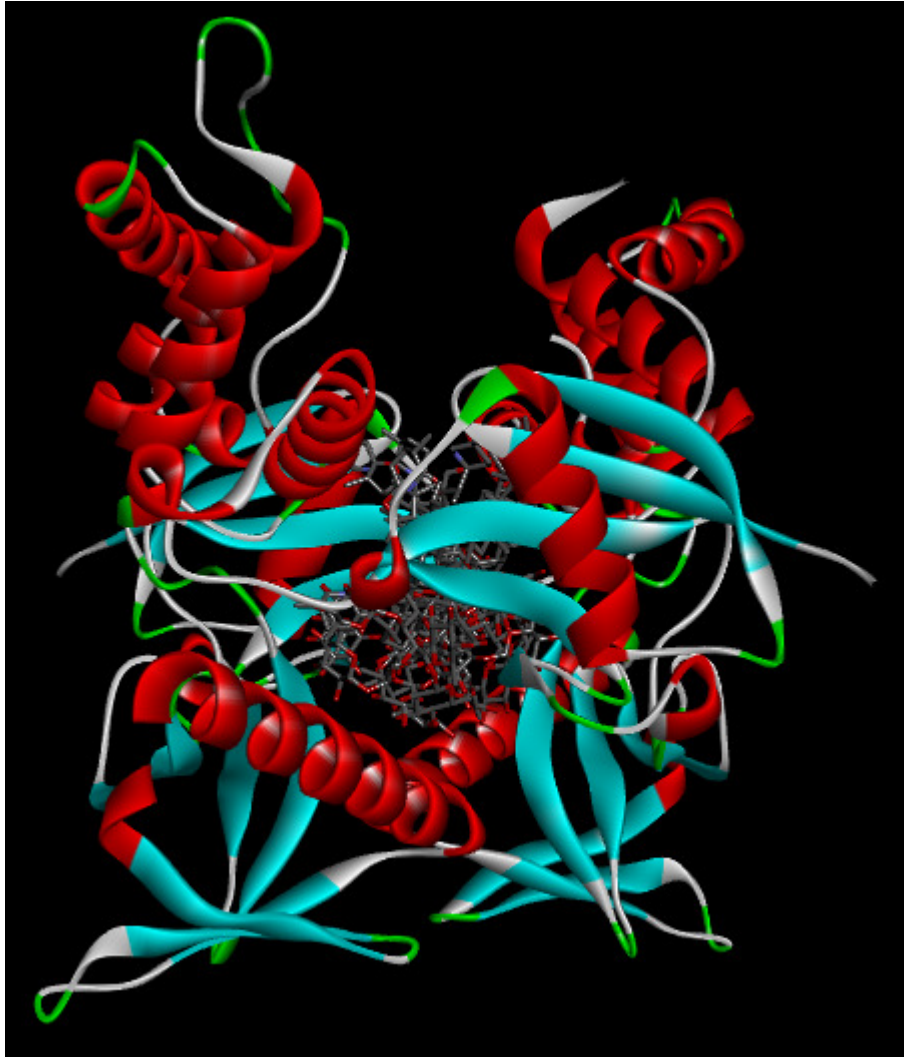

Supplement: Figure S18 [file peerj-07-6012-s018.pdf]

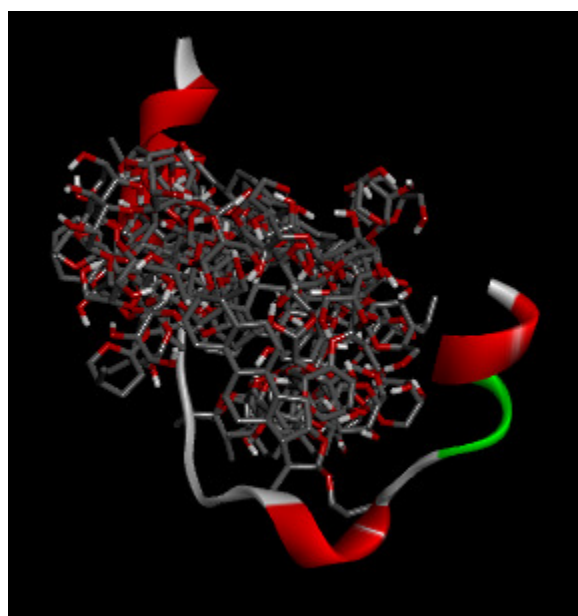

Supplement: Figure S19 [file peerj-07-6012-s019.pdf]

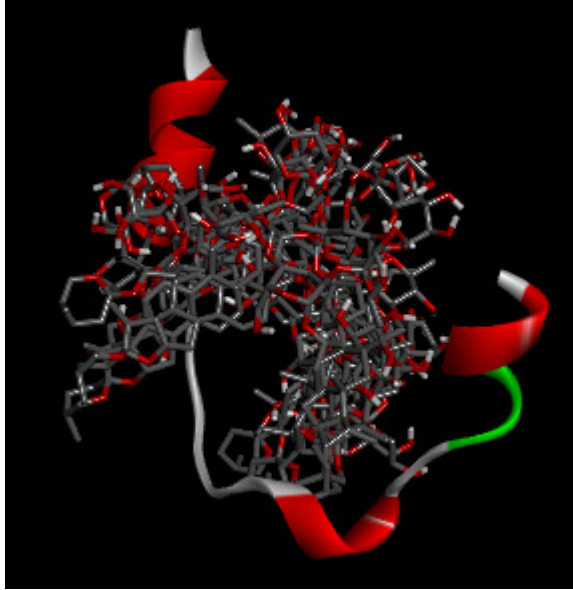

Supplement: Figure S20 [file peerj-07-6012-s020.pdf]

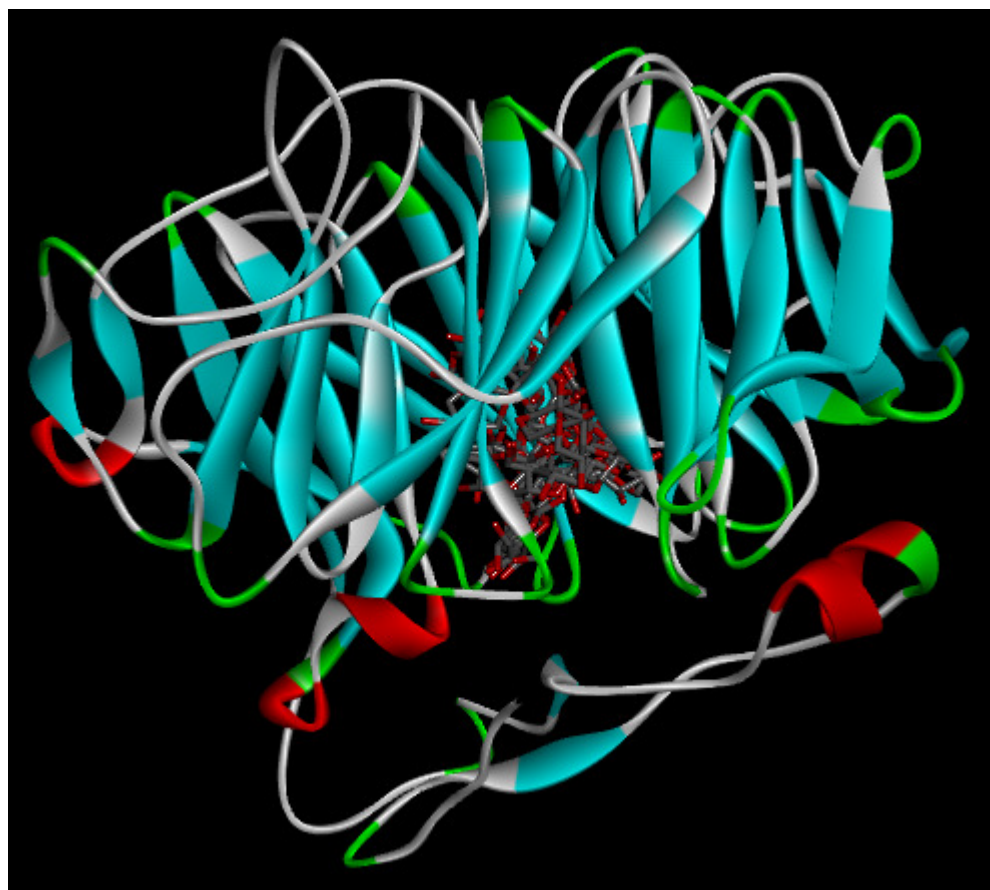

Supplement: Figure S21 [file peerj-07-6012-s021.pdf]
