# Supplementary material for: Steroidal glycoalkaloids from Solanum nigrum target cytoskeletal proteins: an in silico analysis
Source: PeerJ. 2019 Jan 3;7:e6012. doi: 10.7717/peerj.6012 (PMC6321755; doi:10.7717/peerj.6012)
Supplement: Figure S4 [file peerj-07-6012-s004.pdf]

<table>
<tr><th>AutoDock v4.2.6</th><th>AutoDock vina</th><th>iGEMDOCK v2.1</th></tr>
<tr><td colspan="3">Solanidine</td></tr>
<tr><td>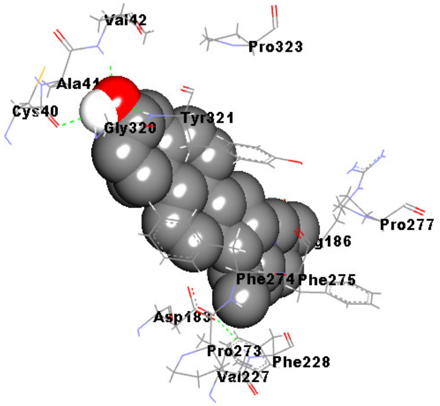
</td><td>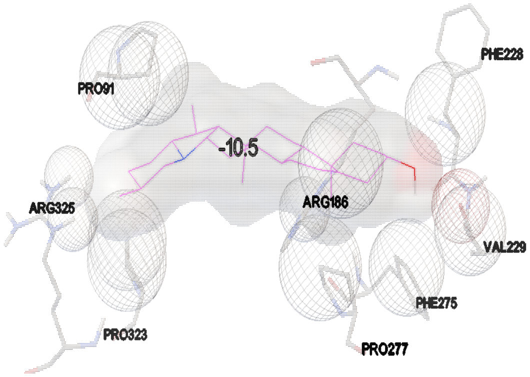
</td><td>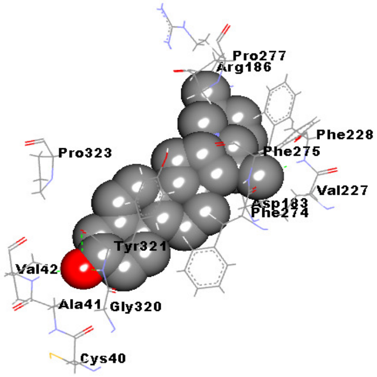
</td></tr>
<tr><td colspan="3">Solasodine</td></tr>
<tr><td>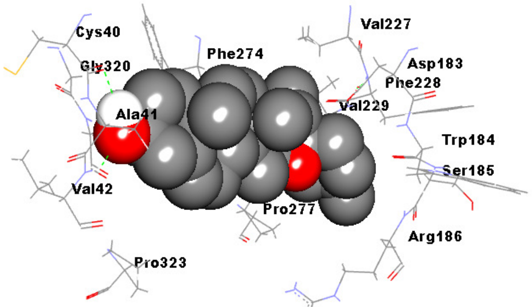
</td><td>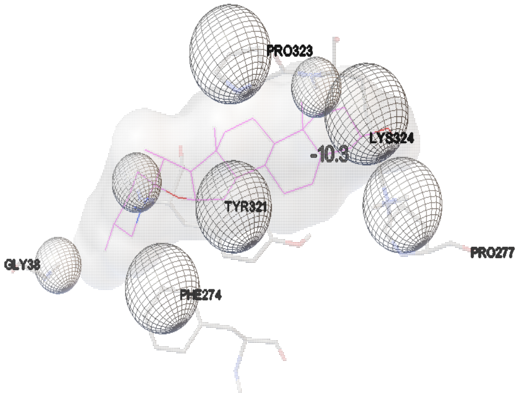
</td><td>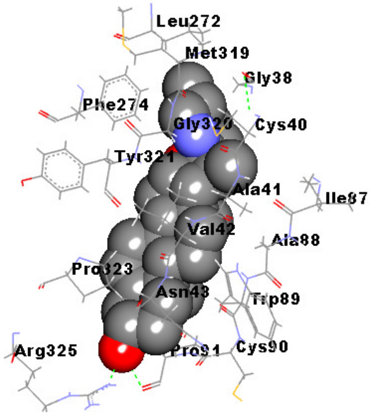
</td></tr>
<tr><td colspan="3">alpha-Solanine</td></tr>
</table>

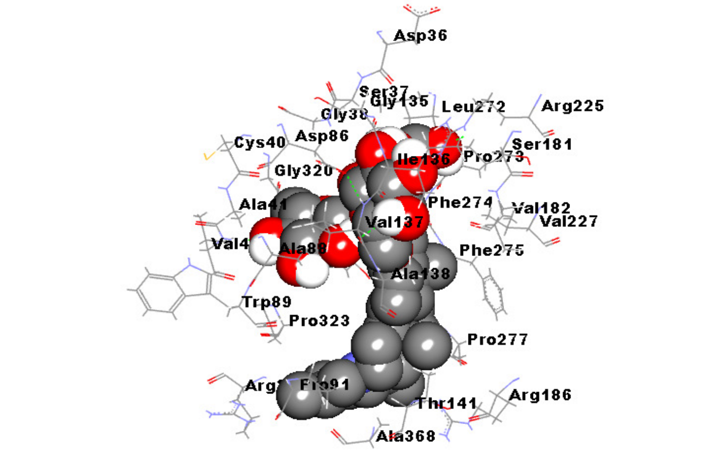

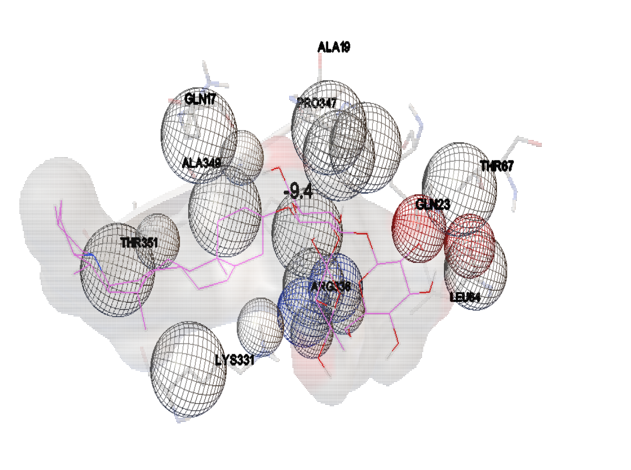

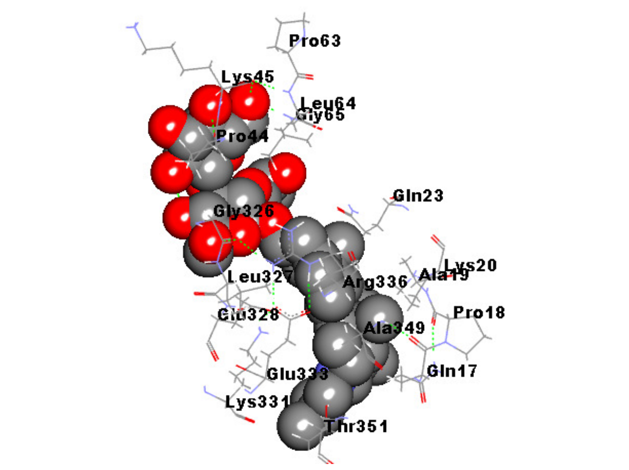

Solasonine

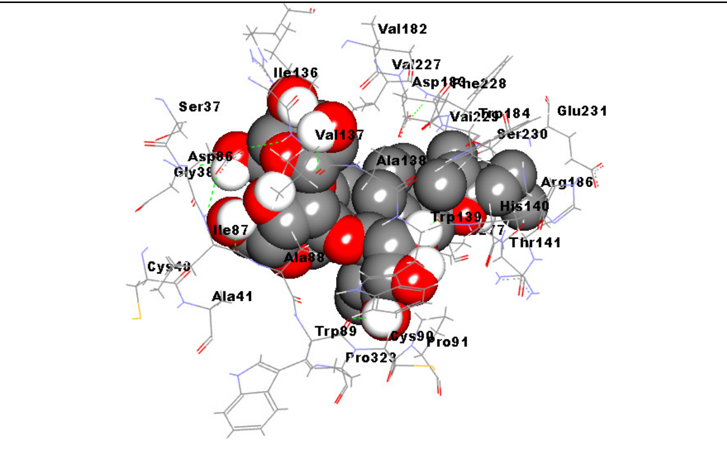

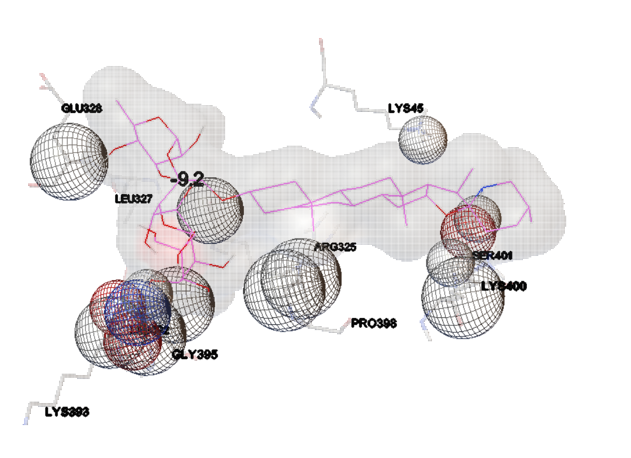

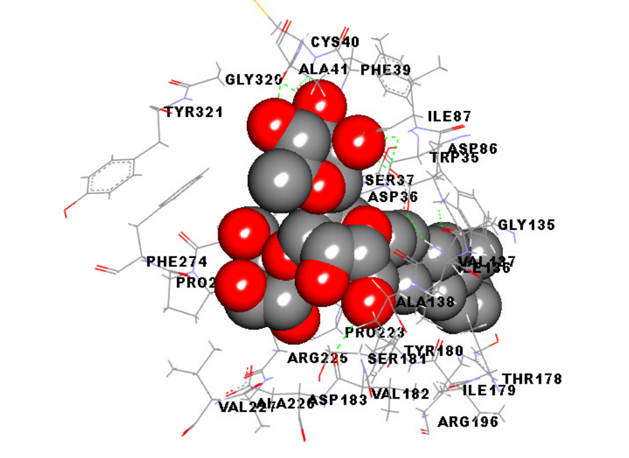

Solamargine

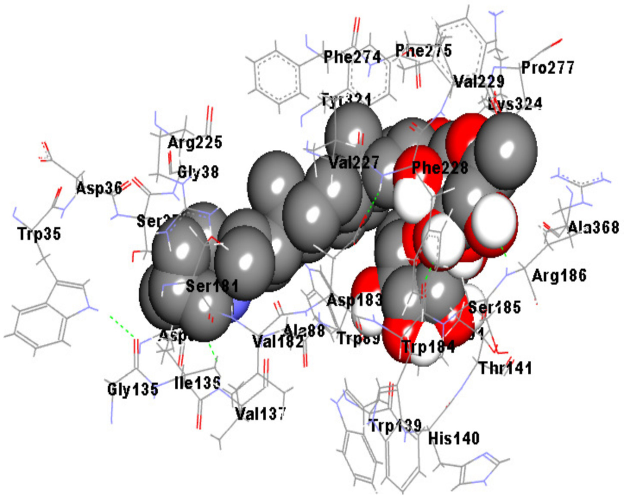

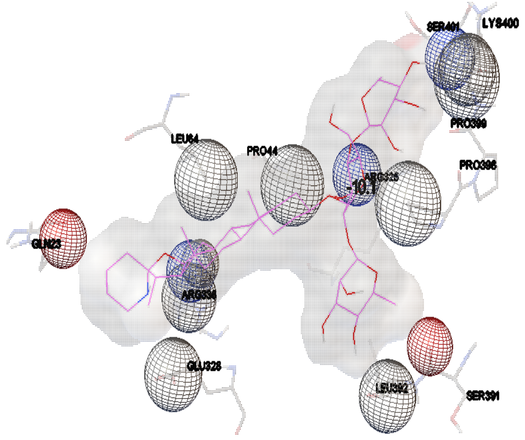

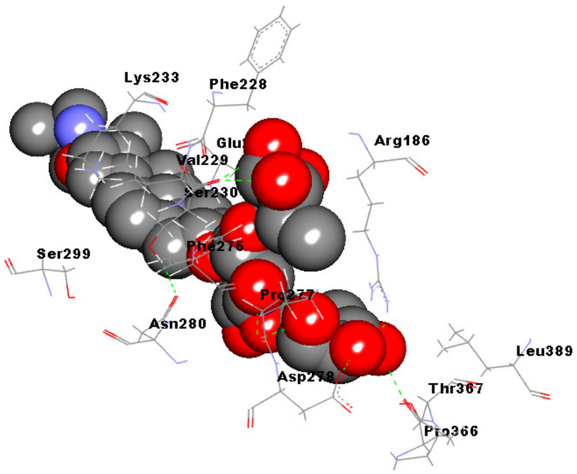

Degalactotigonin

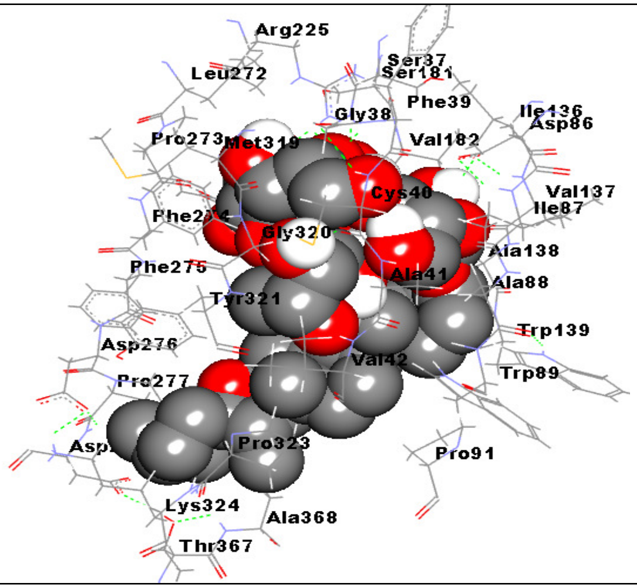

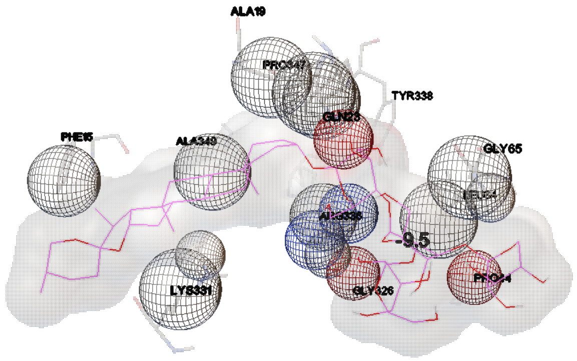

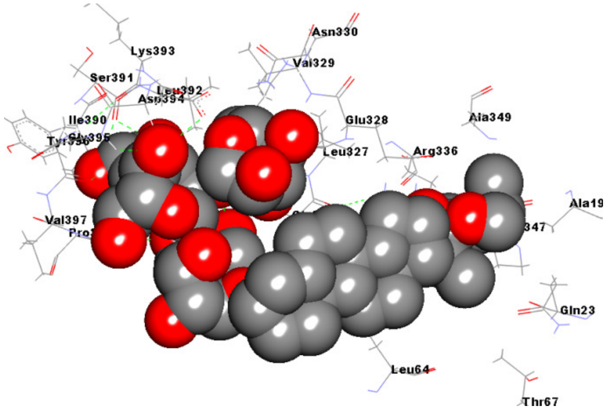

Nigrumnin I

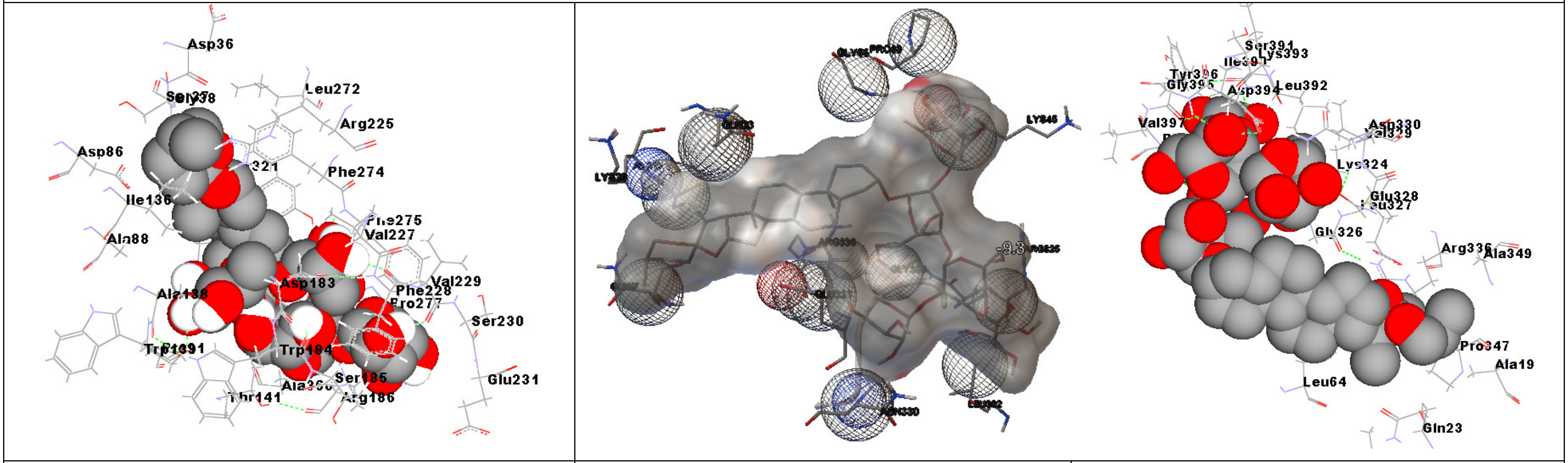

Uttroside B

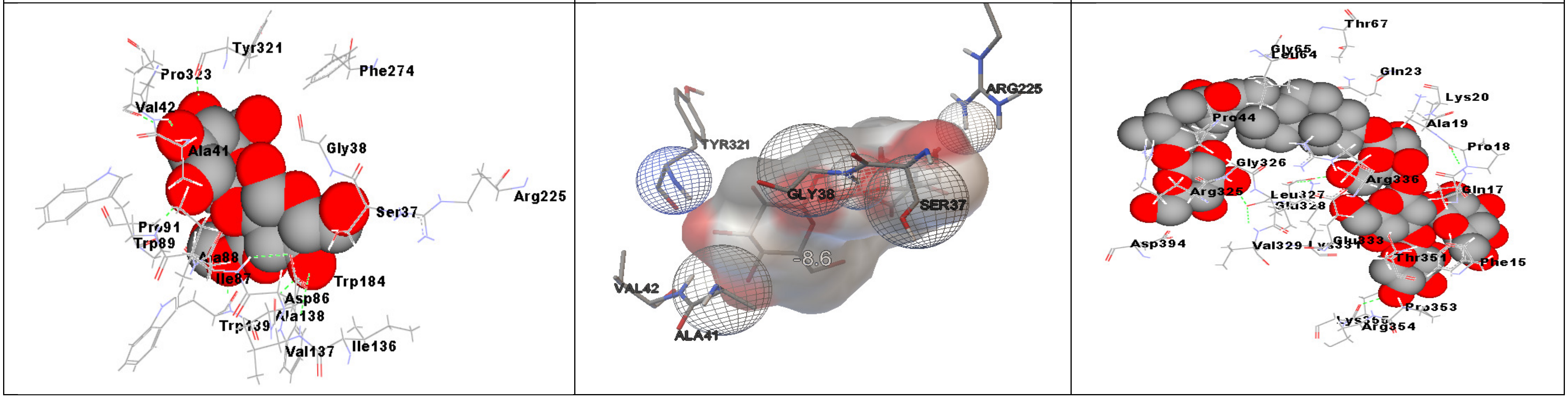

Best docking poses for binding of *S. nigrum* phytoconstituents to Human Coronin-1A
