# Supplementary material for: Steroidal glycoalkaloids from Solanum nigrum target cytoskeletal proteins: an in silico analysis
Source: PeerJ. 2019 Jan 3;7:e6012. doi: 10.7717/peerj.6012 (PMC6321755; doi:10.7717/peerj.6012)
Supplement: Figure S5 [file peerj-07-6012-s005.pdf]

<table>
<tr><th>AutoDock v4.2.6</th><th>AutoDock vina</th><th>iGEMDOCK v2.1</th></tr>
<tr><td colspan="3">Solanidine</td></tr>
<tr>
<td>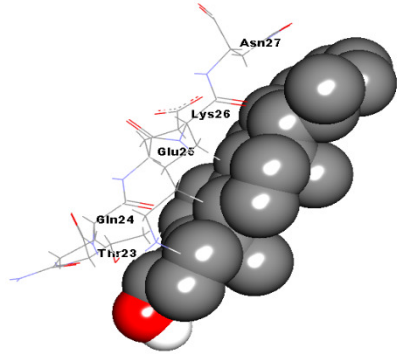
</td>
<td>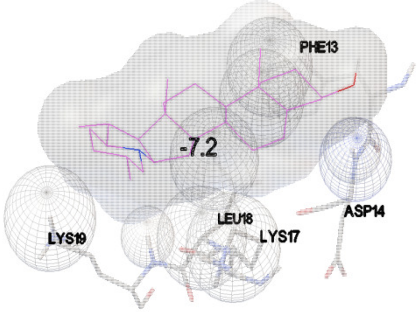
</td>
<td>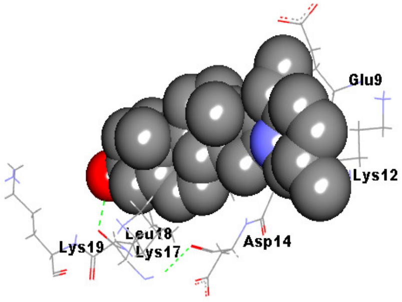
</td>
</tr>
<tr><td colspan="3">Solasodine</td></tr>
<tr>
<td>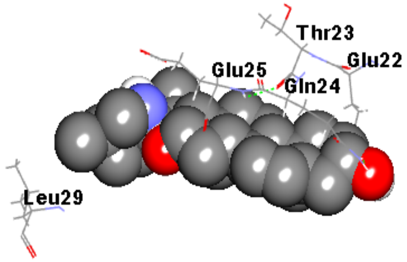
</td>
<td>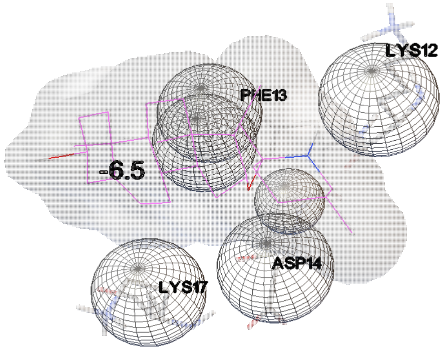
</td>
<td>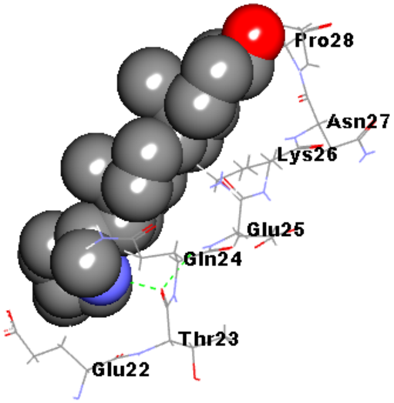
</td>
</tr>
<tr><td colspan="3">alpha-Solanine</td></tr>
</table>

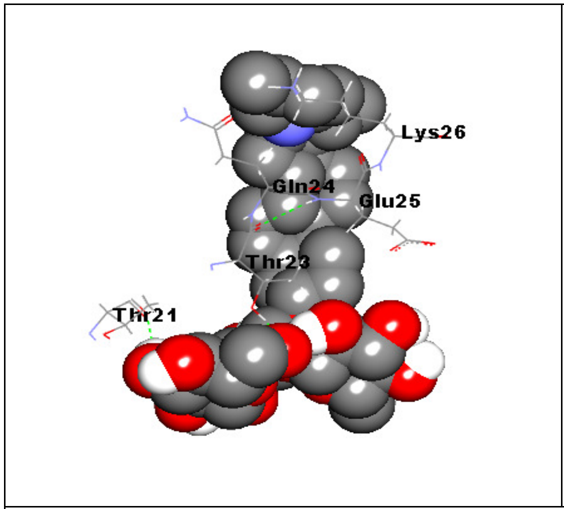

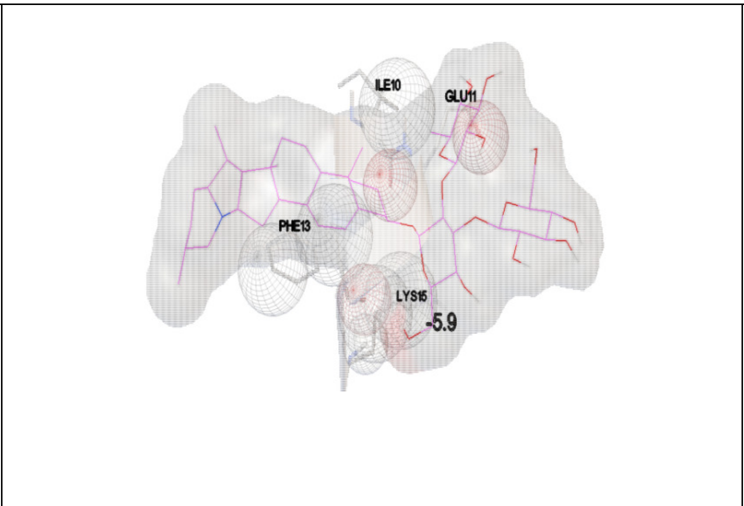

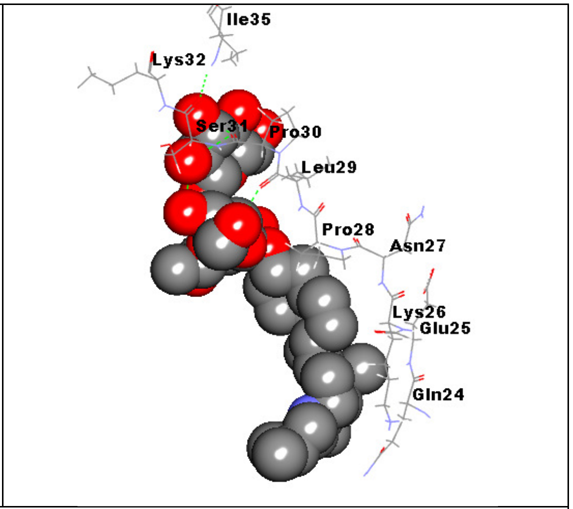

Solasonine

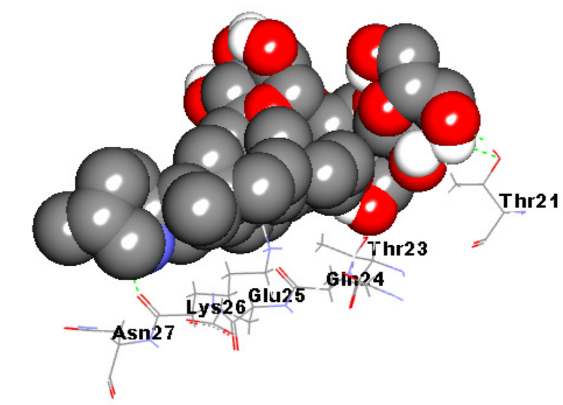

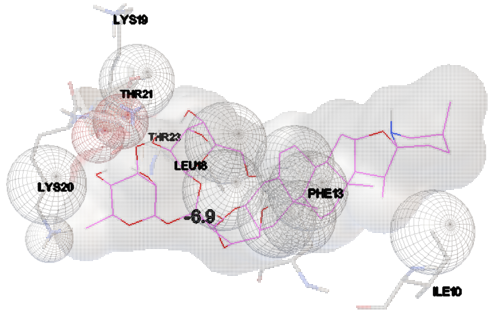

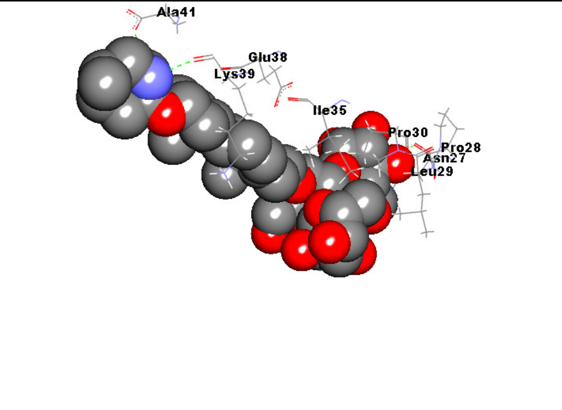

Solamargine

<table>
<tr>
<td>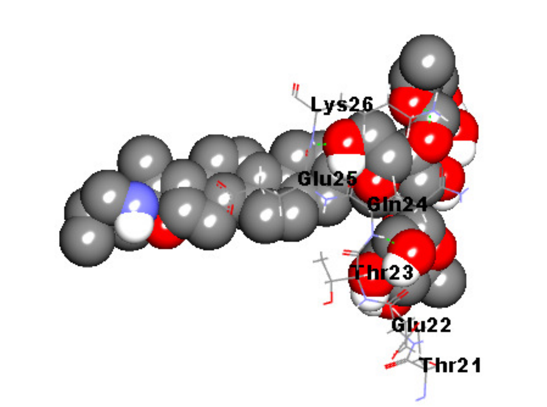
</td>
<td>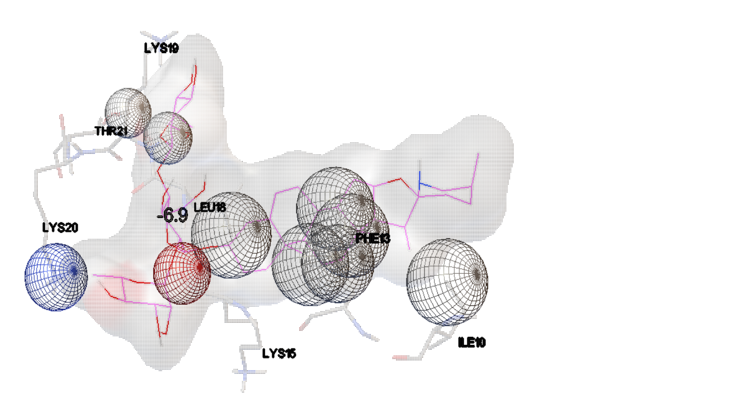
</td>
<td>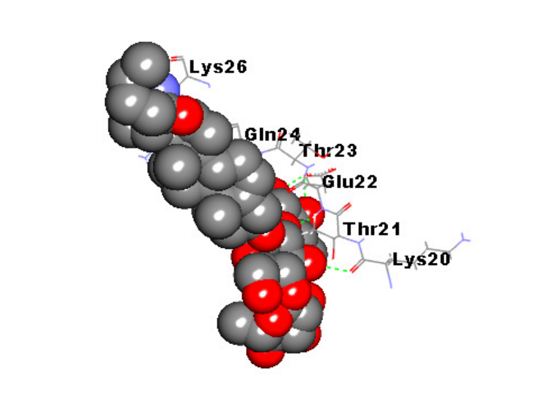
</td>
</tr>
<tr><td colspan="3">Degalactotigonin</td></tr>
<tr>
<td>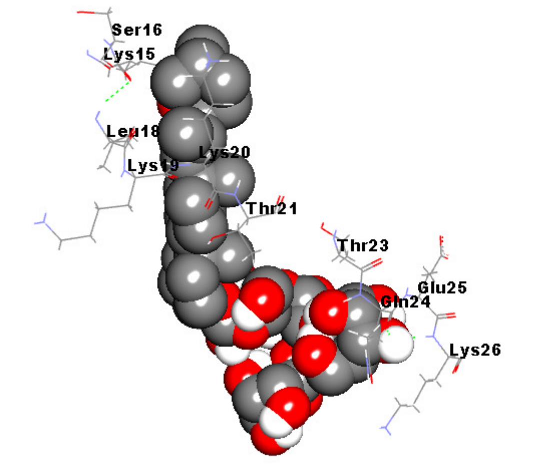
</td>
<td>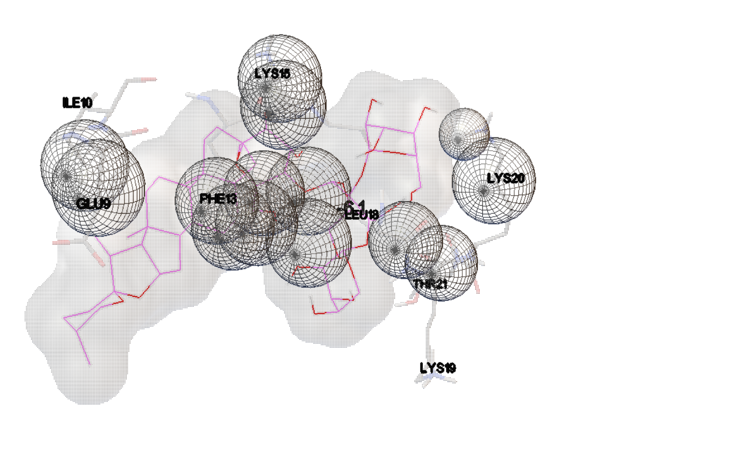
</td>
<td>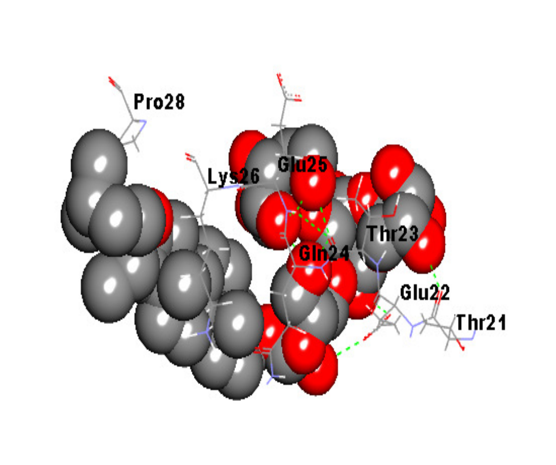
</td>
</tr>
<tr><td colspan="3">Nigrumnin I</td></tr>
</table>

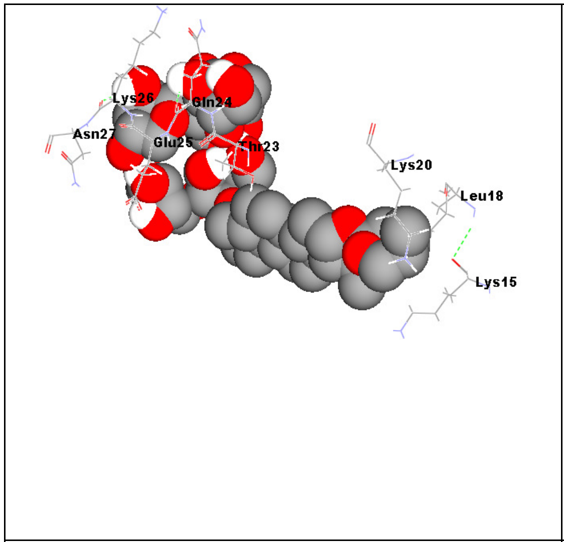

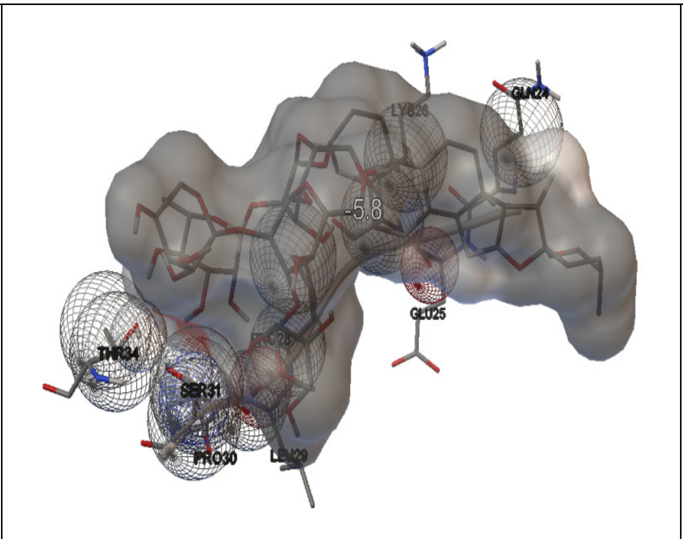

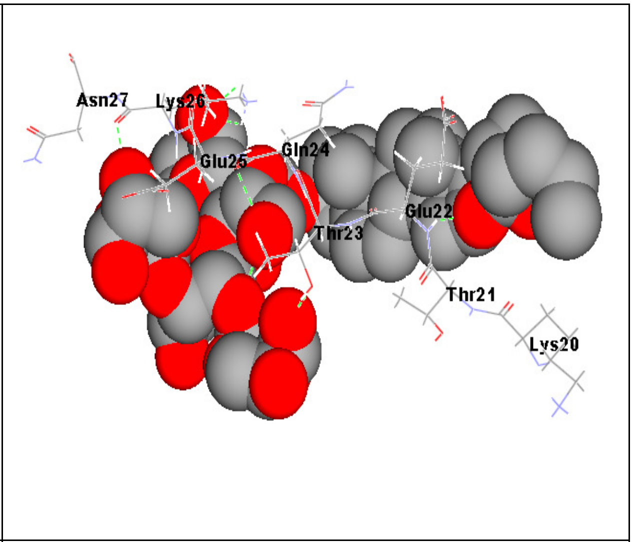

Uttroside B

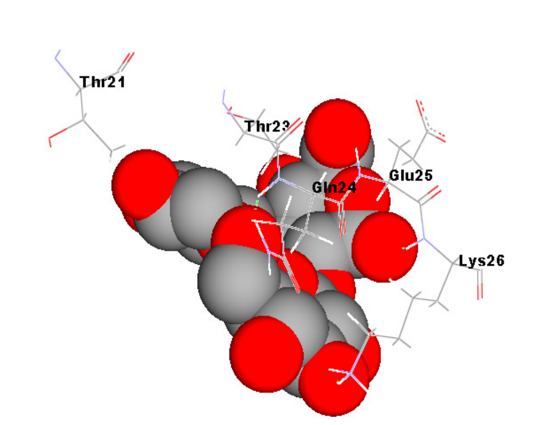

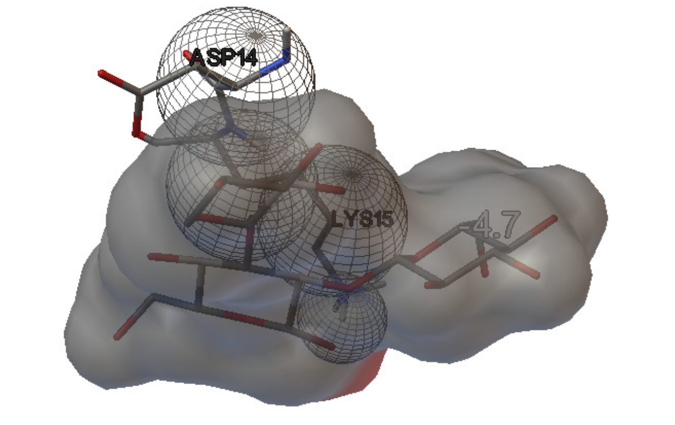

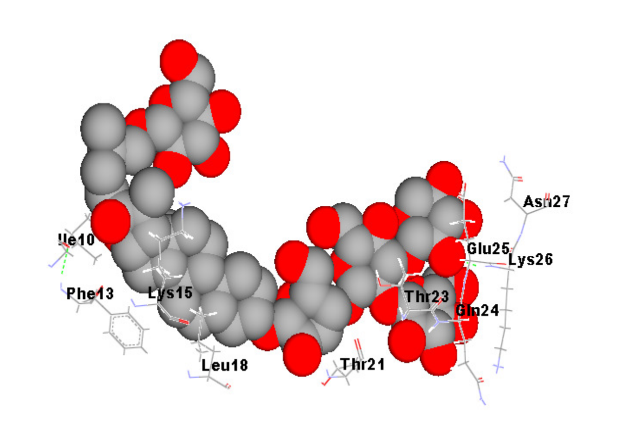

Best docking poses for binding of *S. nigrum* phytoconstituents to Human Thymosin beta-4
