# Supplementary material for: Steroidal glycoalkaloids from Solanum nigrum target cytoskeletal proteins: an in silico analysis
Source: PeerJ. 2019 Jan 3;7:e6012. doi: 10.7717/peerj.6012 (PMC6321755; doi:10.7717/peerj.6012)
Supplement: Figure S6 [file peerj-07-6012-s006.pdf]

A. Vimentin		
**AutoDock v4.2.6**	**AutoDock vina**	**iGEMDOCK v2.1**
**Acetate**		
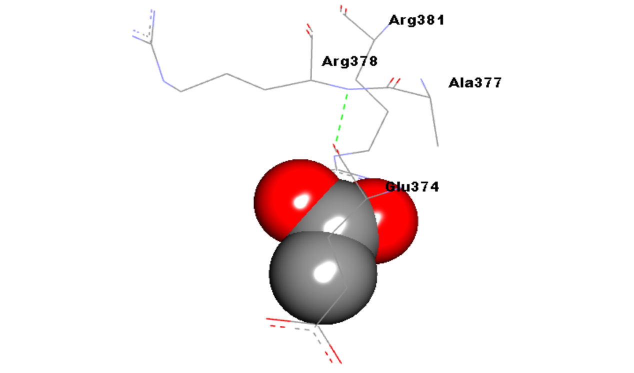	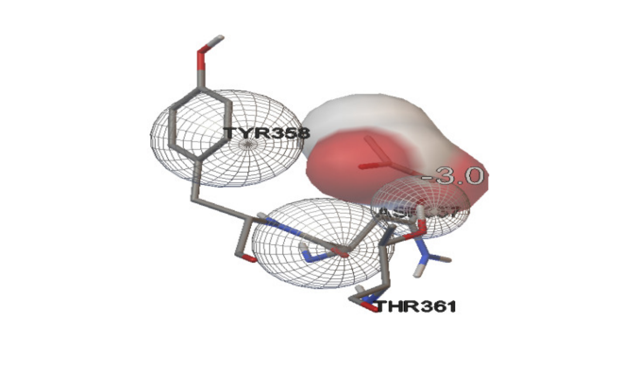	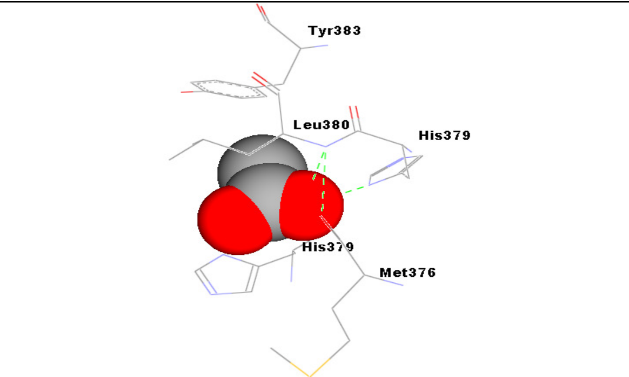
IAAs: Arg381, Arg378, Ala377, Glu374	IAAs: Tyr358, Asn657, Thr361	IAAs: Tyr383, Leu380, His379, Met376
**ATP**		
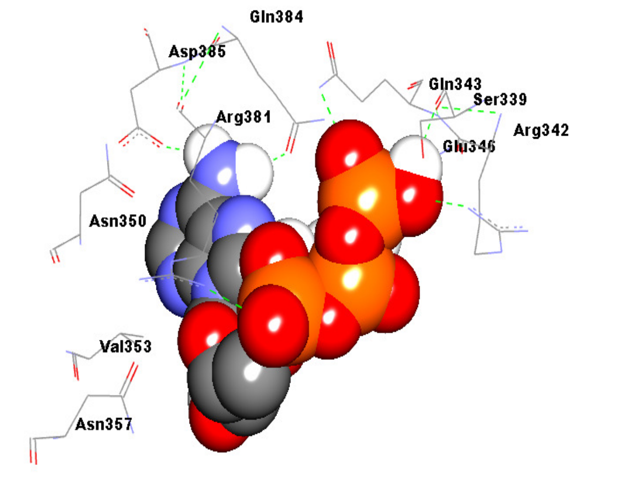	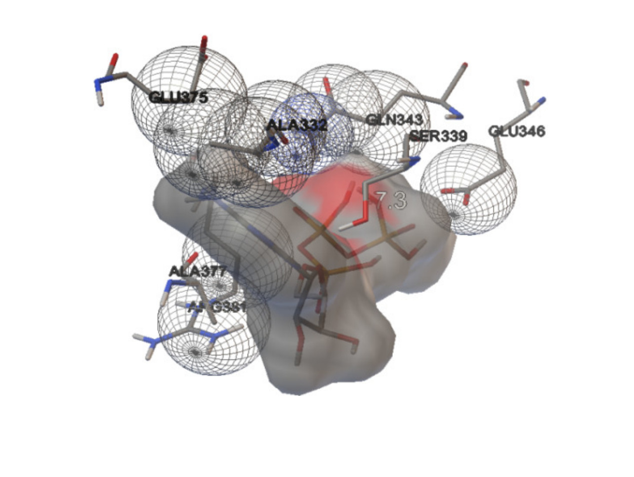	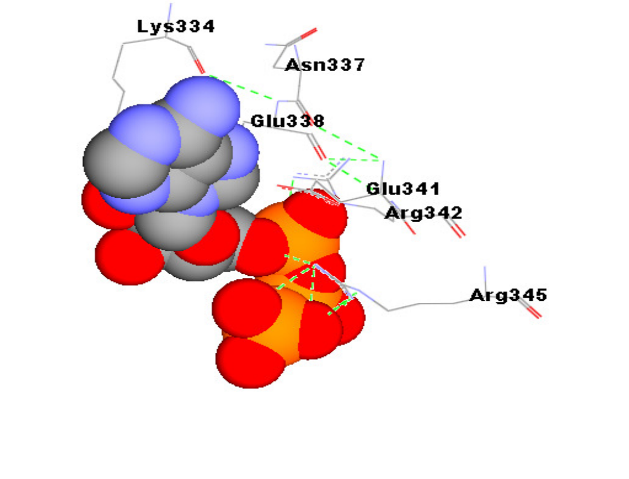
IAAs: Gln384, Gln343, Ser339, Glu346, Arg342, Glu345, Asn357, Val353, Asn350, Arg381, Asp335	IAAs: Glu375, Ala332, Gln343,Ser339,Glu346, Ala377, Arg381	IAAs: Lys334, Asn337, Glu338, Glu341, Arg342, Arg345
**N-acetyl glucosamine**		

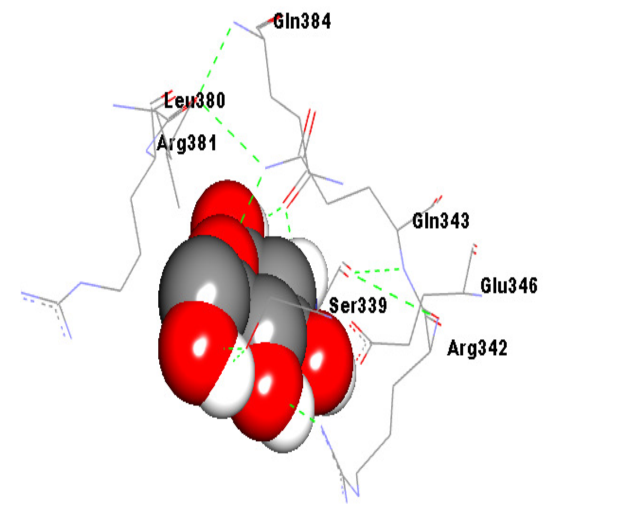	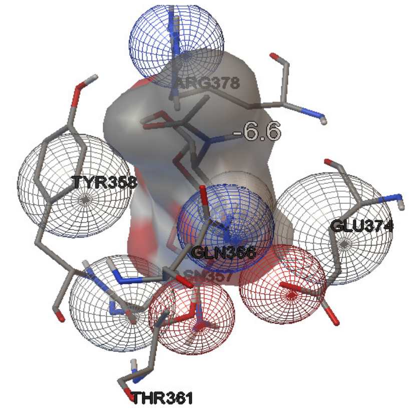	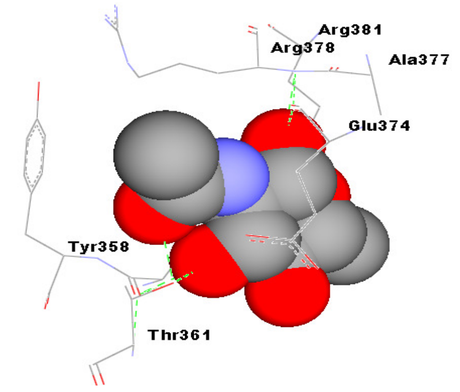
IAAs: Arg381, Leu380, Gln384, Gln343, Glu346, Ser339, Arg342	IAAs:Arg378, Tyr358, Gln366, Glu374, Asn357, Thr361	IAAs: Arg381, Arg378, Ala377, Tyr358, Glu374, Thr361

**B. Gelsolin**	
**AutoDock vina**	**iGEMDOCK v2.1**
**Phosphatidylinositol (4,5)-bisphosphate**	

<table>
<tr>
<td>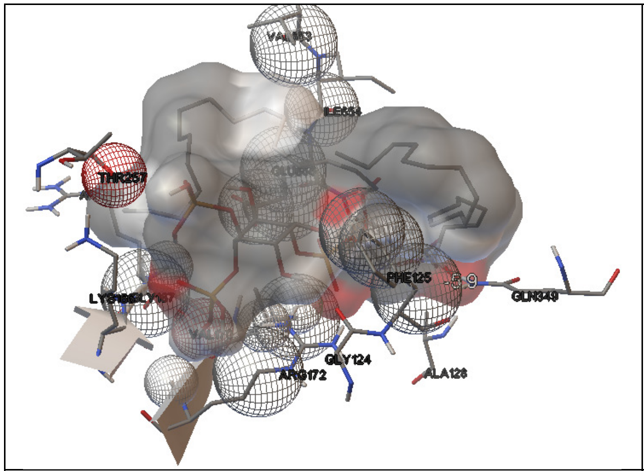
</td>
<td>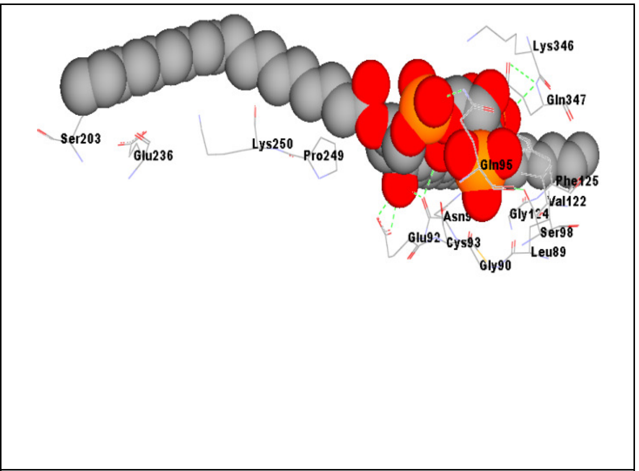
</td>
</tr>
<tr>
<td>IAAs: Val653, Thr257, Glu655, Ileu654, Glu656, Lys166, Val170, Gly167, Arg172, Ala128, Gly124, Phe125, Gln349, Val653, Gly124</td>
<td>IAAs: Asn91, Glu92, Cys93, Gly90, Gln95, Leu89, Ser98, Gly124, Val122, Phe125, Gln347, Lys346, Pro249, Lys250, Glu236, Ser103</td>
</tr>
<tr>
<td colspan="2">Phosphatidylinositol-4-monophosphate</td>
</tr>
<tr>
<td>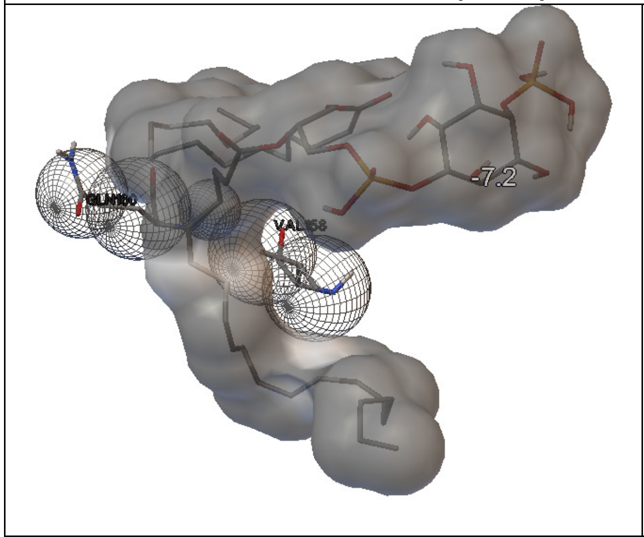
</td>
<td>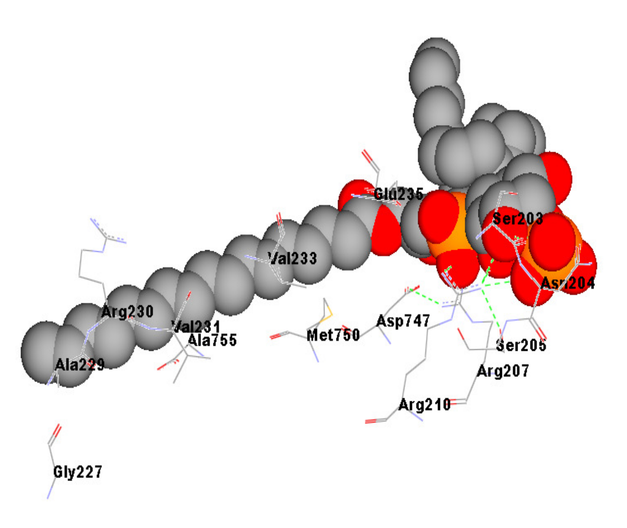
</td>
</tr>
</table>

IAAs: Gln160, Val158	IAAs: Gly227, Ala229, Arg230, Val231, Ala755, Val233, Met750, Asp747, Glu235, Arg210, Arg207, Ser205, Ser203, Asn204, Ala755
**AutoDock v4.2.6**	**iGEMDOCK v2.1**
**ATP**	
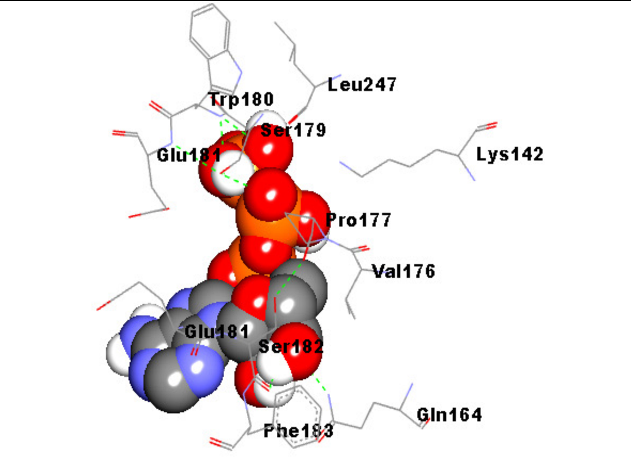	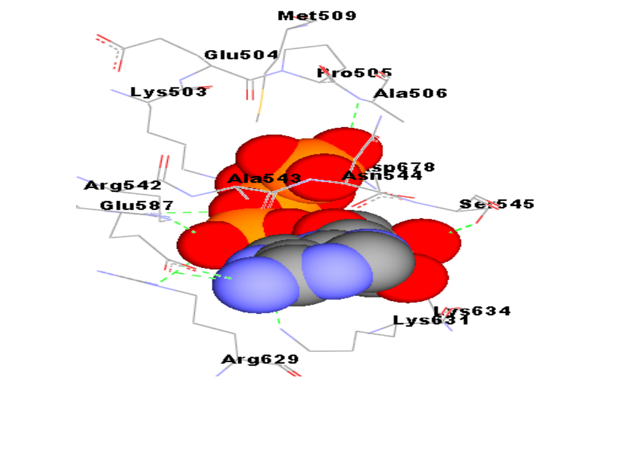
IAAs: Trp180, Leu247, Ser179, Lys142, Pro177, Val176, Gln164, Phe183, Glu181, Ser182	IAAs: Met509, Glu504, Pro505,Ala506,Lys503, Asp678, Asn544, Ser545,Lys634, Lys631, Arg629, Glu587, Arg542,, Ala543

**C. Villin**	
**AutoDock vina**	**iGEMDOCK v2.1**
**Phosphatidylinositol (4,5)-bisphosphate**	

<table>
<tr><td>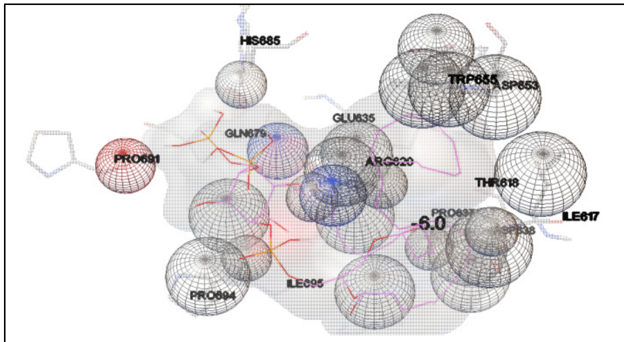
</td><td>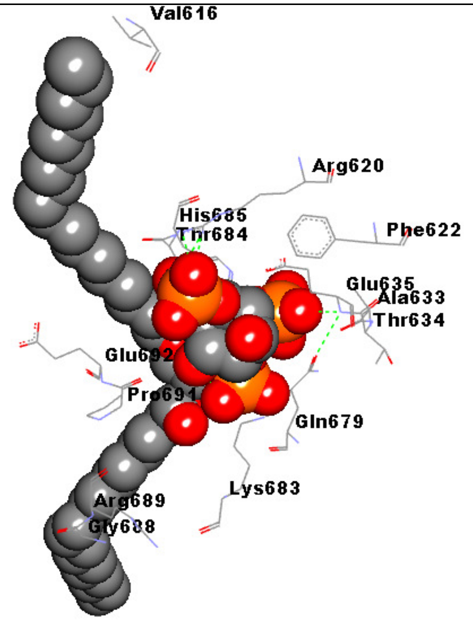
</td></tr>
<tr><td>IAAs: His685, Glu679, Pro691, Pro694, Gln679, Trp655, Asp653, Thr618, Ile617, Arg620, Ile695, Asp638, Pro637</td><td>IAAs: His685, Glu692, Pro694, Gln679, Thr684, Arg620, Arg689, Gly688, Lys683, Val616, Glu635, Ala633, Thr634, Phe622</td></tr>
<tr><td colspan="2">Phosphatidylinositol-4-monophosphate</td></tr>
<tr><td>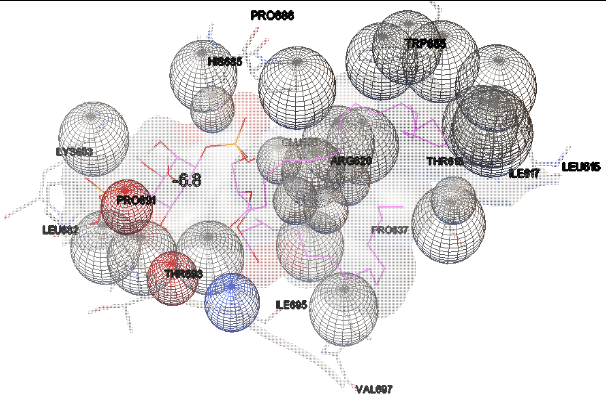
</td><td>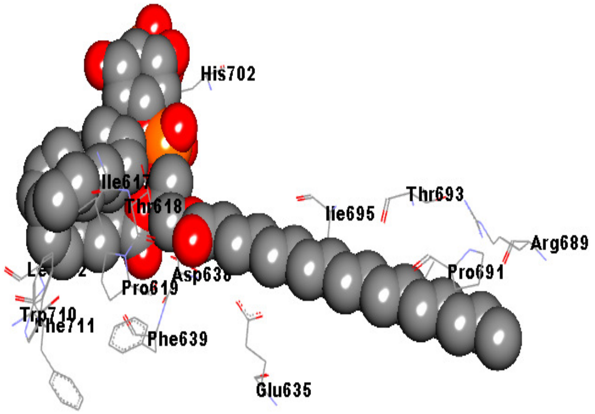
</td></tr>
</table>

<table>
<tr><td colspan="2">IAAs: Lys683, Leu682, Pro691, Thr693, Ile695, His685, Pro686, Glu635, Thr655, Arg620, Pro637, Thr618, Ile617, Leu615, Val697</td><td>IAAs: His702, Leu712, Phe711, Trp710, Thr648, Pro619, Arg689, Thr693, Leu682, Glu635, Phe639, Pro691, Asp638, Thr618, Thr693, Arg689, Ile695</td></tr>
<tr><td colspan="3">Sulfate</td></tr>
<tr><td colspan="2">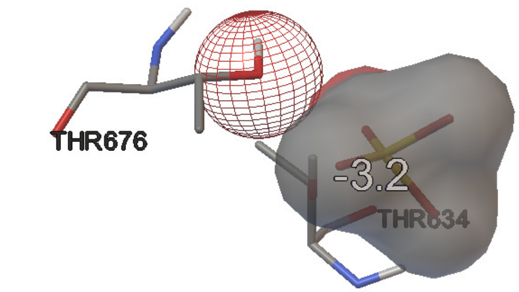
</td><td>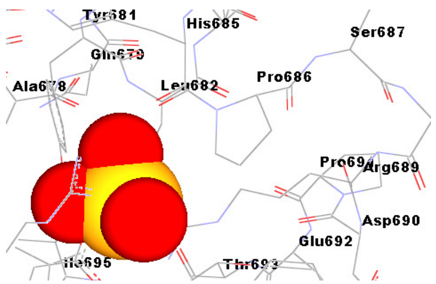
</td></tr>
<tr><td colspan="2">IAAs: Thr634, Thr676</td><td>IAAs: Arg620, Asp653, Tyr681, Arg689</td></tr>
<tr><td>AutoDock v4.2.6</td><td>AutoDock vina</td><td>iGEMDOCK v2.1</td></tr>
<tr><td colspan="3">Acetate</td></tr>
<tr><td>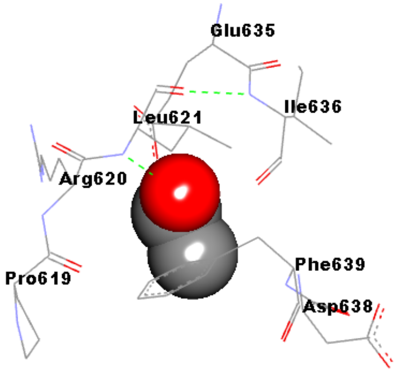
</td><td>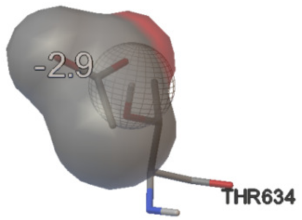
</td><td>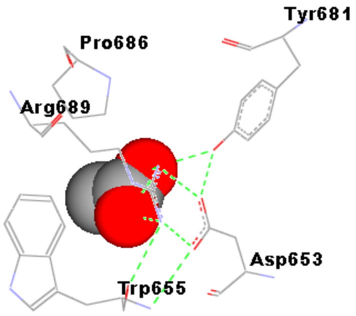
</td></tr>
<tr><td>IAAs: Pro619, Arg620, Leu621, Glu635, Ile636, Phe639, Asp538</td><td>IAAs: Thr634</td><td>IAAs: Arg689, Asp653, Tyr681, Pro686, Trp655</td></tr>
<tr><td colspan="3">ATP</td></tr>
<tr><td></td><td></td><td></td></tr>
</table>

<table>
<tr>
<td>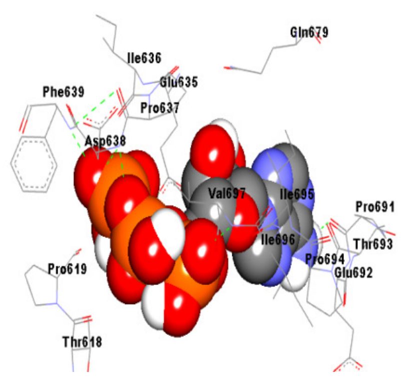
</td>
<td>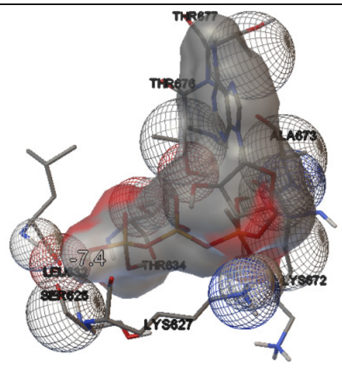
</td>
<td>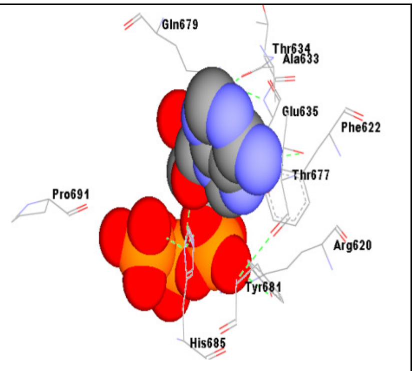
</td>
</tr>
<tr>
<td>IAAs: Phe639, Asp638, Pro637, Ile636, Glu535, Gln679, Val697, Ile695, Ile696, Pro694, Thr693, Glu692, Pro619, Thr618</td>
<td>IAAs: Thr634, Thr676, Ala673, Thr677, Lys672, Lys627, Ser625, Leu632</td>
<td>IAAs: Pro691, Gln679, Thr634, Ala633, Glu635, Phe622, Thr677, Arg620, Tyr681, His685</td>
</tr>
</table>

**D. Coronin-1A**	
**AutoDock vina**	**iGEMDOCK v2.1**
**Phosphatidylinositol (4,5)-bisphosphate**	

<table>
<tr>
<td>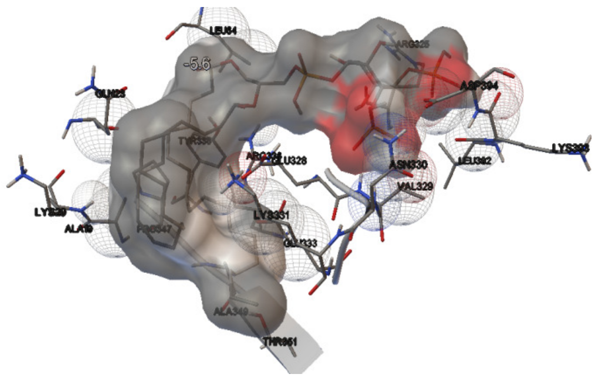
</td>
<td>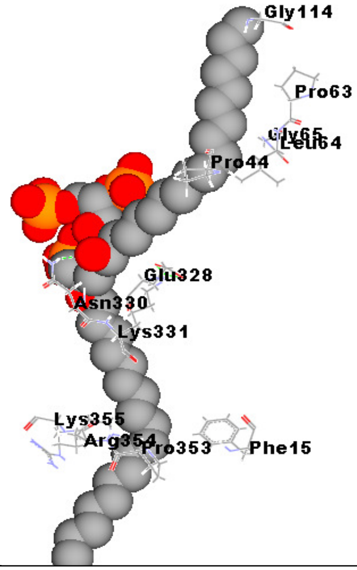
</td>
</tr>
<tr>
<td>IAAs: Arg 325, Asp394, Leu392,Lys393, Val329, Leu64,Arg336,gln23, Tyr338, Lys20,Pro347,Thr351, Asn330, Val329, Glu328, Lys331, Glu333, Ala19, Pro347, Ala349, Thr351, Lys392</td>
<td>IAAs:Gly114,Pro63,Gly65,Leu64,Pro44,Glu328,Asn 330, Lys331, Lys355, Arg354, Pro353, Phe15</td>
</tr>
<tr>
<td colspan="2">Phosphatidylinositol-4-monophosphate</td>
</tr>
<tr>
<td>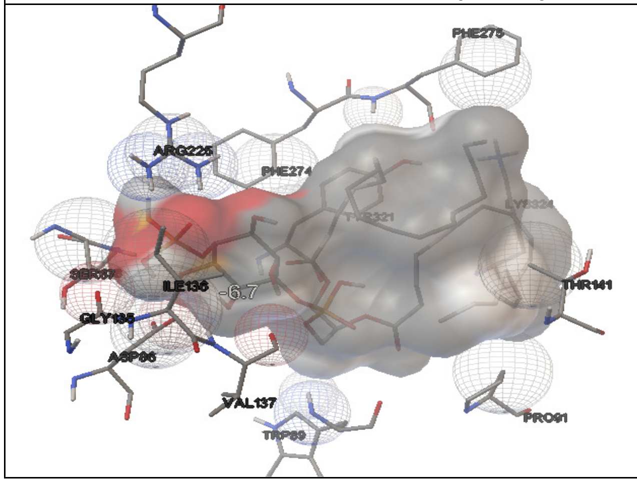
</td>
<td>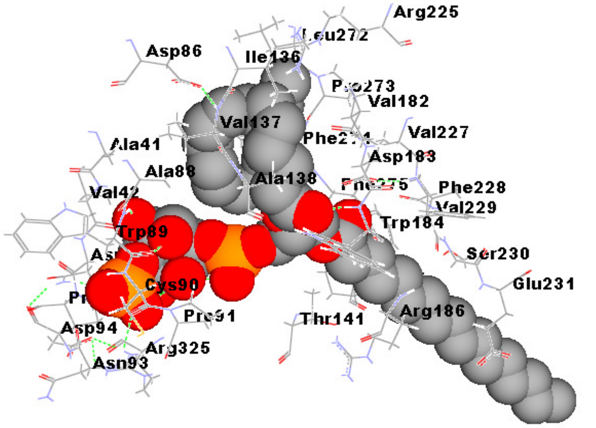
</td>
</tr>
</table>

IAAs: Arg225, Phe274, Tyr321, Lys324, Thr141, Pro91, Trp89, Val137, Ileu136, Asp86, Gly135, Ser37, Phe275	IAAs: Asn43, Trp89, Asp94,Asp183, Trp184, Pro91, Val137, Arg186, Arg225, Ser230, Glu231, Phe274,Phe275, Pro277, Pro44, Ala41, Asp86, Ileu136, Leu272, Pro273, Val182, Val227, Phe228, Cys90,Arg325,Asn93,Val42,Ala138,Ala88,Val229, Thr141,Val42	
**AutoDock v4.2.6**	**AutoDock vina**	**iGEMDOCK v2.1**
**ATP**		
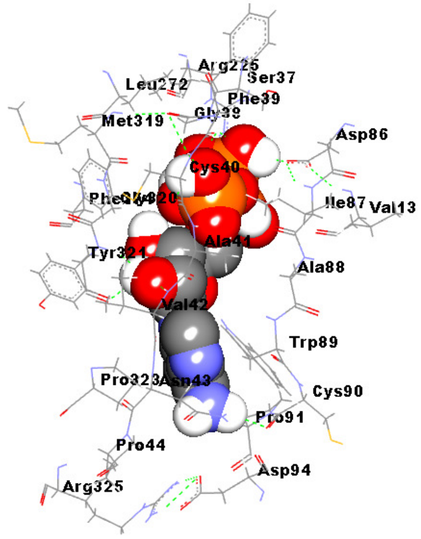	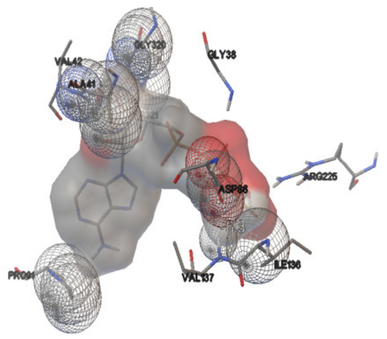	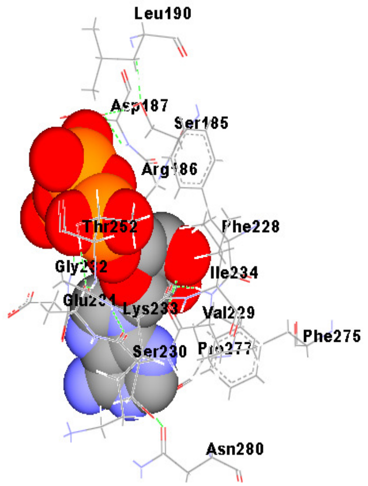
IAAs: Arg226, Ser37, Phe39, Leu272, Met319, Gly38, Asp86, Ile97, Ala98, Trp89, Cys90, Pro91, Asp94, Arg325, Pro44, Pro323, Tyr321, Gly320, Phe275,	IAAs: Arg225, Ile136, Val137, Pro91, Asp86, Tyr321,Gly320, Val42, Ala41, Gly38	IAAs: Leu190, Asp187, Ser185, Val229, Gly232,Lys233, Pro277, Asn280, Arg186, Phe228, Ser230, Glu231,Phe275, Ile234, Thr252,
**AutoDock v4.2.6**	**AutoDock vina**	
**Acetate**		

<table>
<tr><td>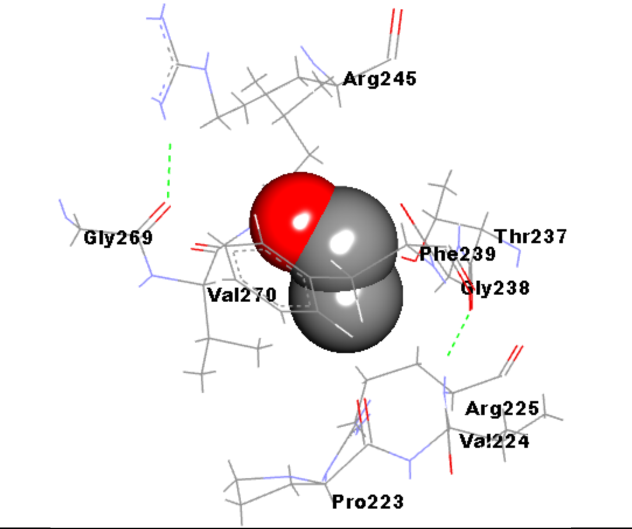
</td><td>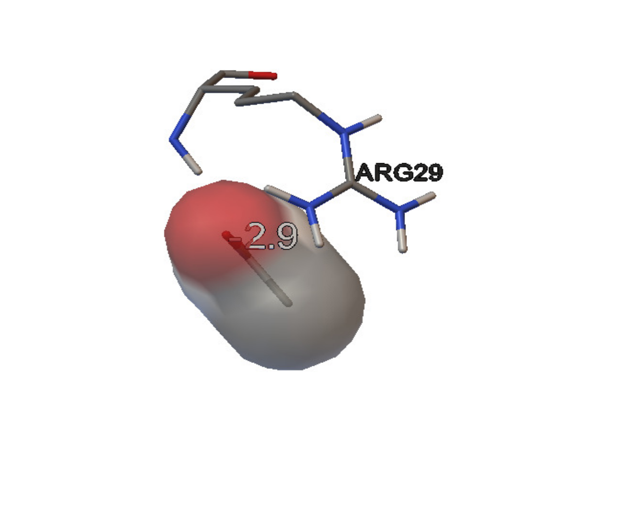
</td></tr>
<tr><td>IAAs:Gly269, Val270, Pro223, Val224, Arg226,Gly238, Phe239, Thr237, Arg245</td><td>IAAs: Arg229</td></tr>
<tr><td colspan="2">AutoDock vina</td></tr>
<tr><td>Sulfate</td><td>Zinc</td></tr>
<tr><td>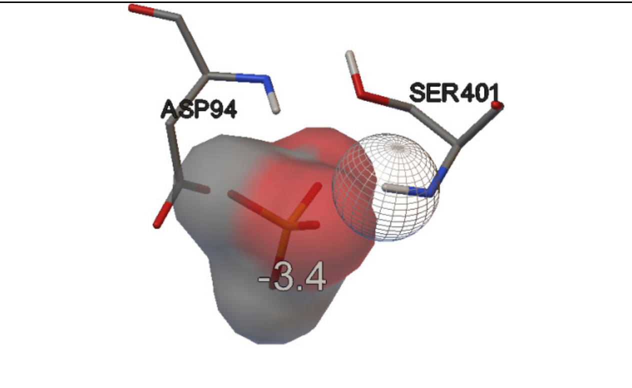
</td><td>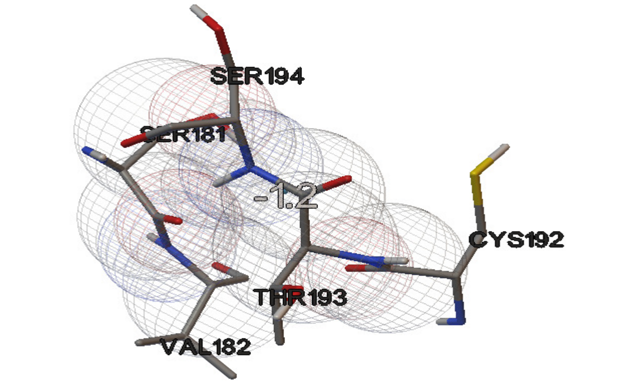
</td></tr>
<tr><td>IAAs: Asp94, Ser401</td><td>IAAs: Ser194, Ser181, Thr193, Val182, Cys192</td></tr>
</table>

<table>
<tr><th colspan="3">E. Thymosin β-4</th></tr>
<tr><th>AutoDock v4.2.6</th><th>AutoDock vina</th><th>iGEMDOCK v2.1</th></tr>
<tr><th colspan="3">ATP</th></tr>
<tr><td>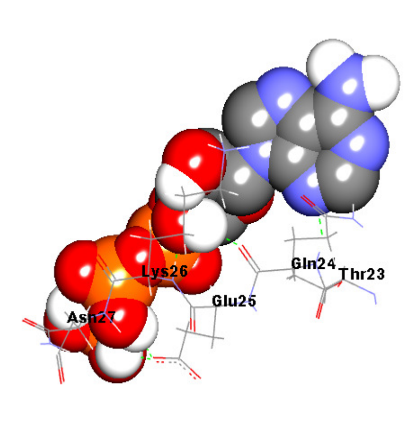
</td><td>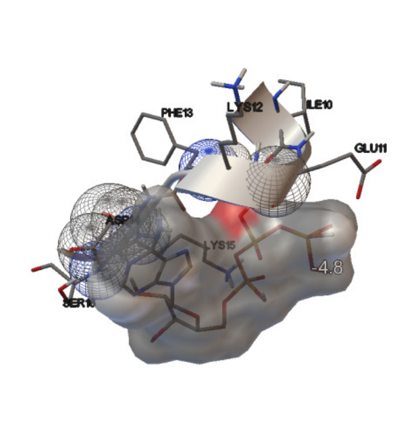
</td><td>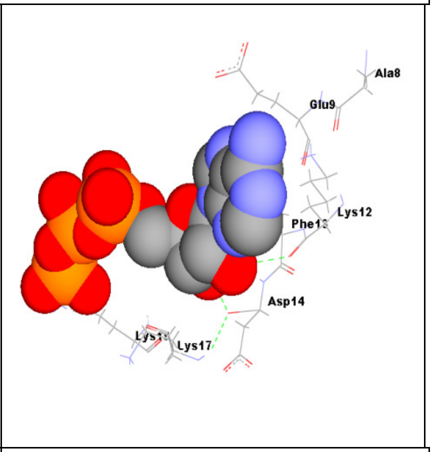
</td></tr>
<tr><td>IAAs: Asn227, Lys26, Glu25, Gln42, Thr23</td><td>IAAs: Phe13,Lys12, Ile10, Glu11,Asp14, Lys15, Ser16</td><td>IAAs: Lys12, Lys13, Lys17, Lys19, Asp14, Glu9, Ala8, Phe13</td></tr>
<tr><th colspan="3">AutoDock vina</th></tr>
<tr><th colspan="3">Calcium</th></tr>
<tr><td colspan="3">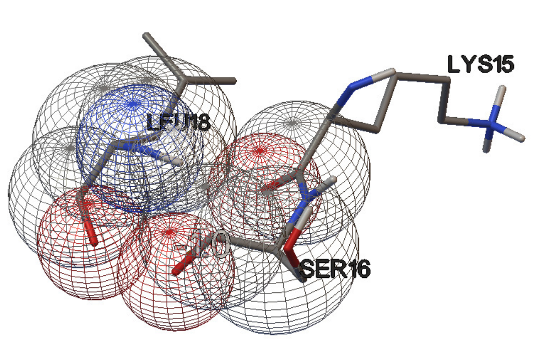
</td></tr>
<tr><td colspan="3">IAAs: Leu18, Ser16, Lys15</td></tr>
</table>

<table>
<tr><th colspan="3">F. Ezrin</th></tr>
<tr><th colspan="3">AutoDock vina</th></tr>
<tr><th colspan="3">Sulfate</th></tr>
<tr><td colspan="3">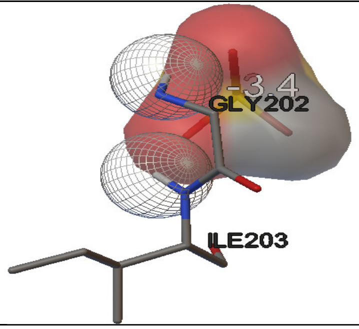
</td></tr>
<tr><td colspan="3">IAAs: Gly202, Ile203</td></tr>
<tr><th>AutoDock v4.2.6</th><th>AutoDock vina</th><th>iGEMDOCK v2.1</th></tr>
<tr><th colspan="3">ATP</th></tr>
<tr><td>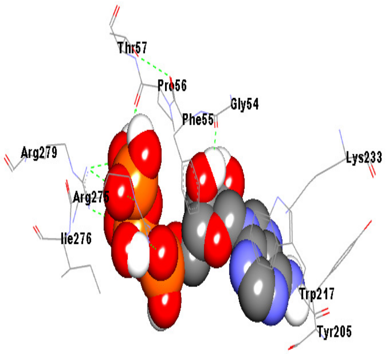
</td><td>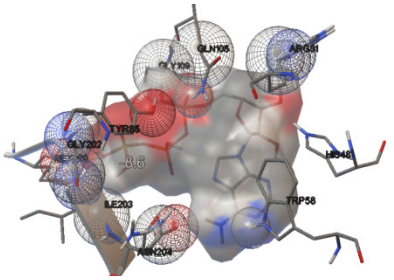</td><td>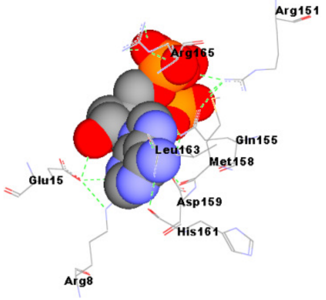
</td></tr>
<tr><td>IAAs: Thr 57, Pro56, Phe55, Gly54, Lys233, Trp217, Tyr205, Ile276, Arg275, Arg279</td><td>IAAs: Gly202, Tyr85, Gly109, Gln105, Arg31, His481, Trp58, Asn204, Ile203</td><td>IAAs: Arg8, Glu15, Arg165, Arg151, Leu163, Gln155, Met158, Asp159, His161</td></tr>
</table>

**Phosphatidylinositol (4,5)-bisphosphate**	
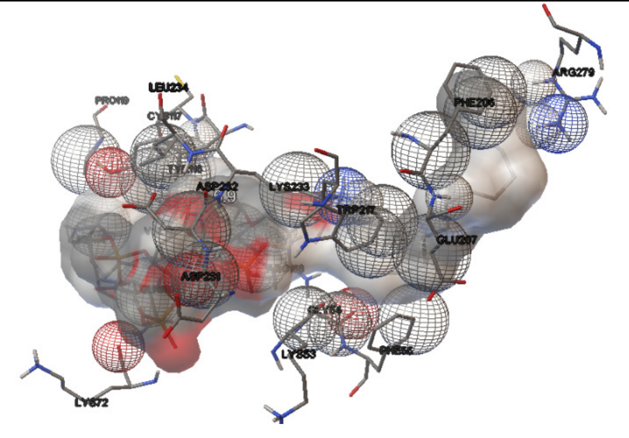 	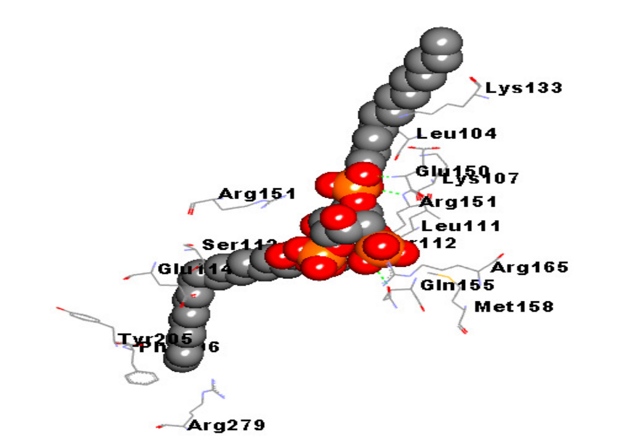
IAAs: Pro119, Leu234, Cys117, Asp231, Asp232, Lys233, Trp217, Gly54, Lys53, Phe55, Glu207, Phe206, Arg279, Lys72	IAAs: Lys133, Leu104,Glu150, Lys107, Arg151, Leu111, Arg165, Gln155, Met158, Arg279, Tyr205, Phe206, Glu114, Ser112, Arg151

**G. Cofilin**
**AutoDock vina**
**Mg**

<table>
<tr><td colspan="2">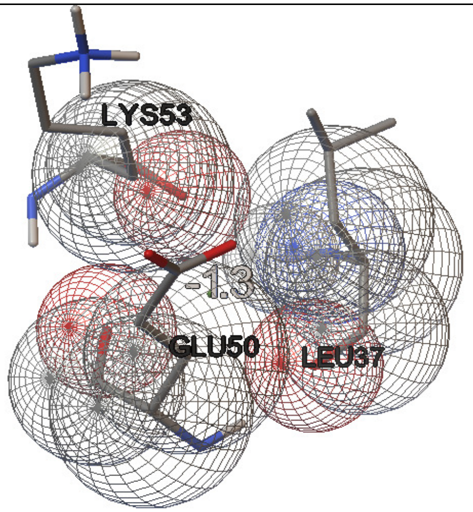
</td></tr>
<tr><td colspan="2">IAAs: Lys53, Glu50, Leu37</td></tr>
<tr><td>AutoDock vina</td><td>iGEMDOCK v2.1</td></tr>
<tr><td colspan="2">ATP</td></tr>
<tr><td>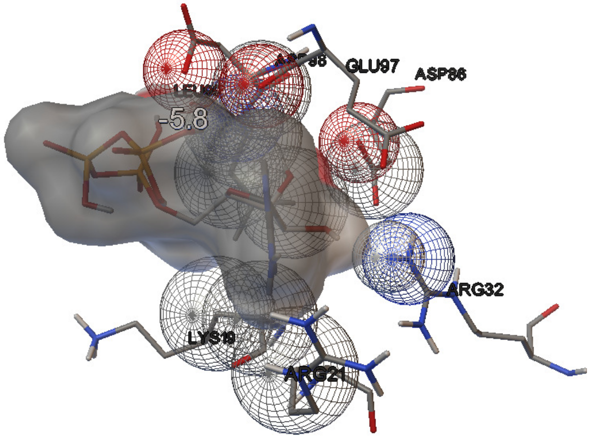
</td><td>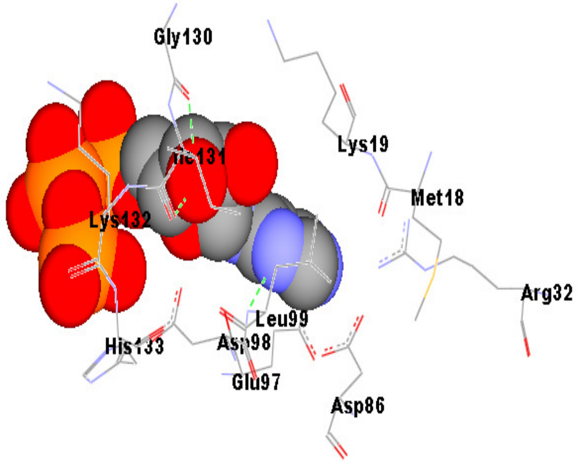
</td></tr>
<tr><td>IAAs: Leu99, Asp98, Glu97, Asp86, Lys19, Arg21, Arg32</td><td>IAAs: Gly130, Lys19, Met18, Arg32, Asp86, Leu99, Asp98, Glu97, Ile131, Lys132, His133</td></tr>
</table>

Best docking poses for binding of physiological ligands to (A) Vimentin (B) Gelsolin (C) Villin (D) Coronin-1A (E) Thymosin β-4 (F) Ezrin (G) Cofilin. The poses were generated using AutoDock v4.2.6, AutoDock vina and iGEMDOCK v2.1.
