# Supplementary material for: Steroidal glycoalkaloids from Solanum nigrum target cytoskeletal proteins: an in silico analysis
Source: PeerJ. 2019 Jan 3;7:e6012. doi: 10.7717/peerj.6012 (PMC6321755; doi:10.7717/peerj.6012)
Supplement: Figure S7 — The poses were generated using AutoDock v4.2.6. [file peerj-07-6012-s007.pdf]

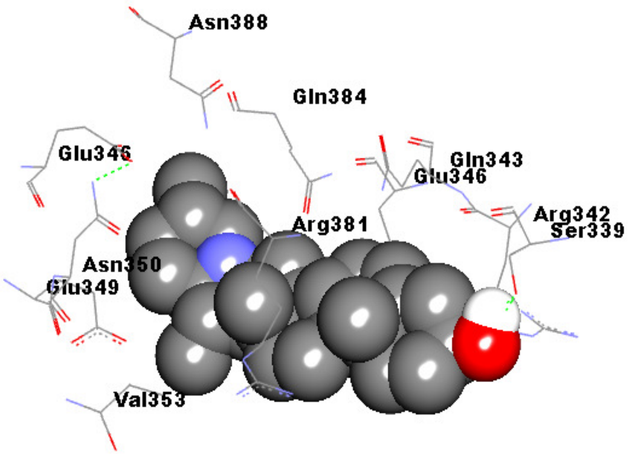
Asn388
Gln384
Glu346
Gln343
Glu346
Arg342
Ser339
Arg381
Asn350
Glu349
Val353

(a)

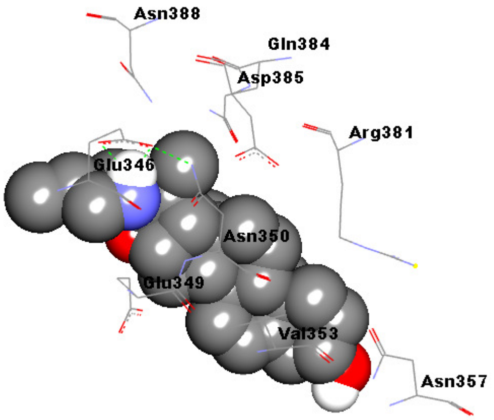
Asn388
Gln384
Asp385
Arg381
Glu346
Asn350
Glu349
Val353
Asn357

(b)

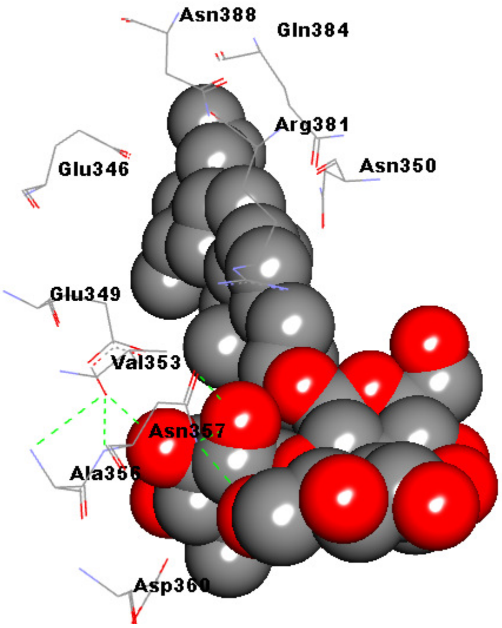
Asn388
Gln384
Arg381
Glu346
Asn350
Glu349
Val353
Asn357
Asp360

(c)

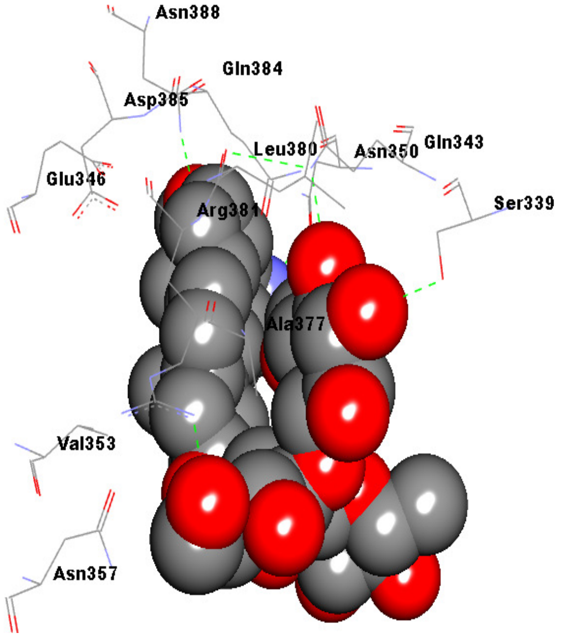
Asn388
Gln384
Asp385
Leu380
Asn350
Gln343
Glu346
Ser339
Arg381
Ala377
Val353
Asn357

(d)

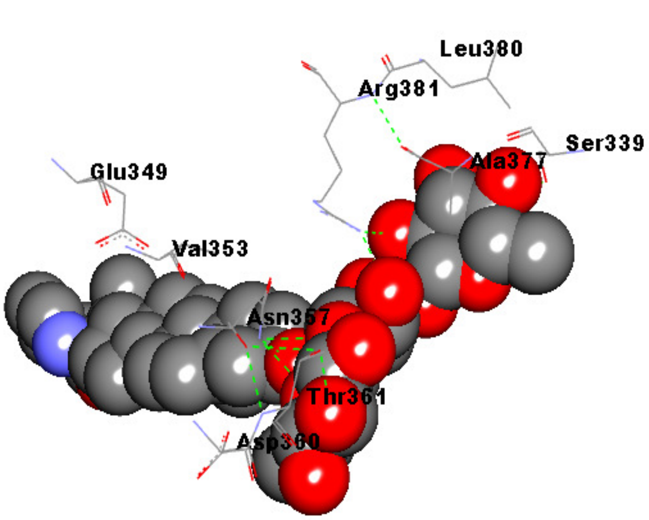

(e)

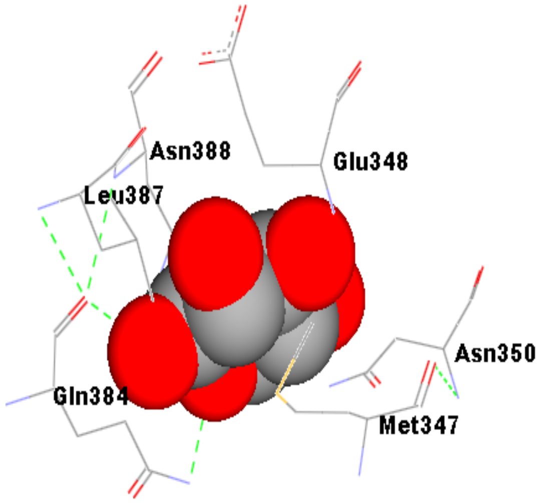

(f)

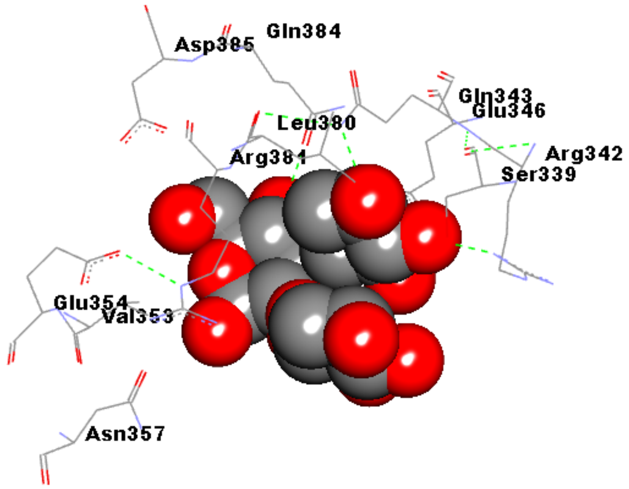

(g)

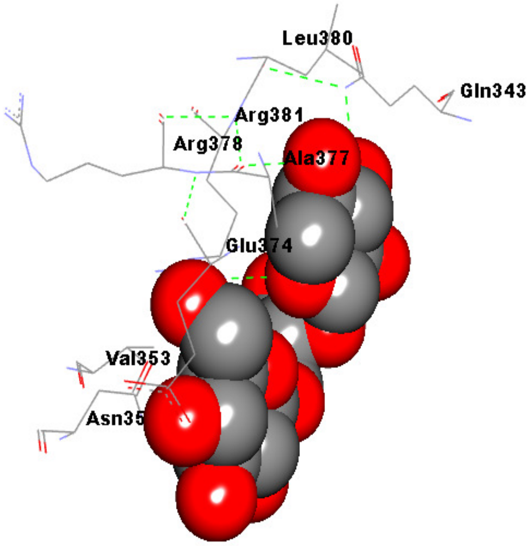

(h)

Best docking poses for binding of (a) solanidine, (b) solasodine, (c) alpha-solanine, (d) solasonine, e) solamargine, (f) degalactotigonin, (g) nigrumnin-I and (h) uttroside B with Vimentin. The poses were generated using AutoDock v4.2.6.
