# Supplementary material for: Steroidal glycoalkaloids from Solanum nigrum target cytoskeletal proteins: an in silico analysis
Source: PeerJ. 2019 Jan 3;7:e6012. doi: 10.7717/peerj.6012 (PMC6321755; doi:10.7717/peerj.6012)
Supplement: Figure S8 — The poses were generated using AutoDock v4.2.6. [file peerj-07-6012-s008.pdf]

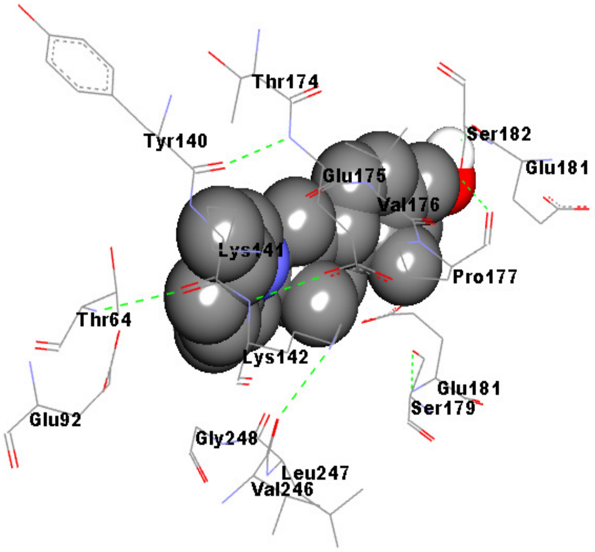
Thr174
Tyr140
Ser182
Glu181
Glu175
Val176
Lys141
Pro177
Thr64
Lys142
Glu181
Ser179
Glu92
Gly248
Leu247
Val246

(a)

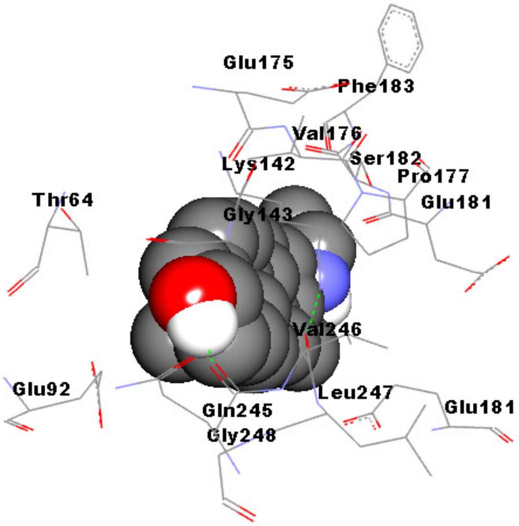
Glu175
Phe183
Val176
Ser182
Lys142
Pro177
Thr64
Glu181
Gly143
Val246
Glu92
Leu247
Glu181
Gln245
Gly248

(b)

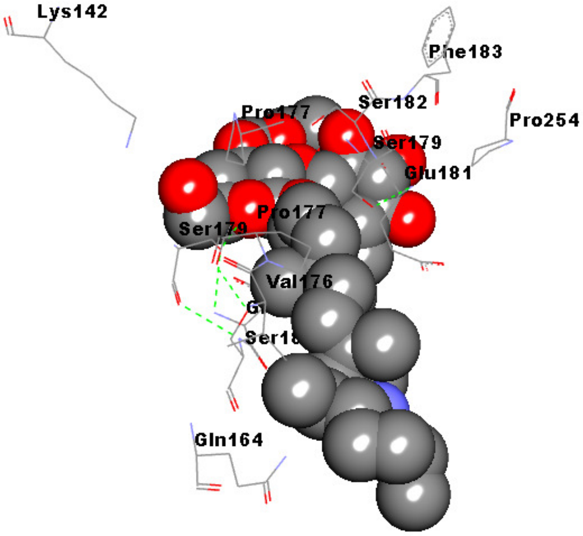
Lys142
Phe183
Ser182
Pro254
Pro177
Ser179
Glu181
Pro177
Ser179
Val176
Gln164

(c)

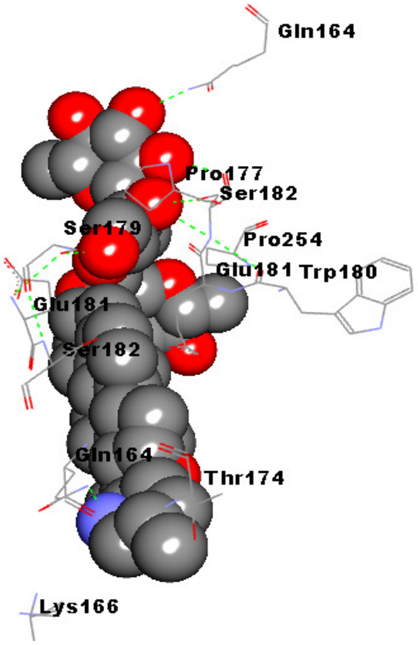
Gln164
Pro177
Ser182
Ser179
Pro254
Glu181
Trp180
Glu181
Ser182
Gln164
Thr174
Lys166

(d)

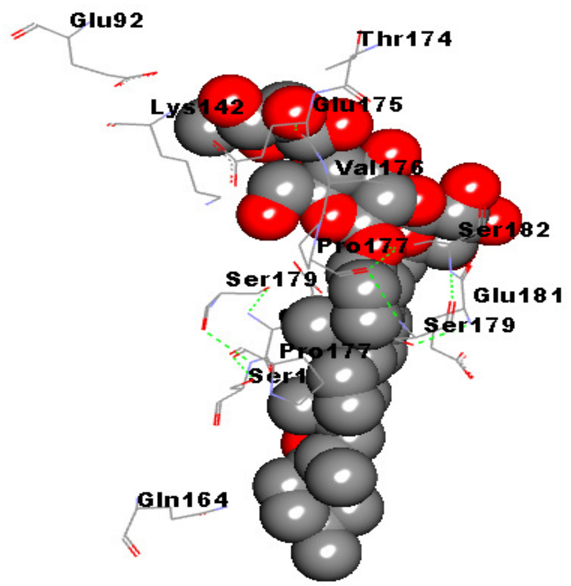

(e)

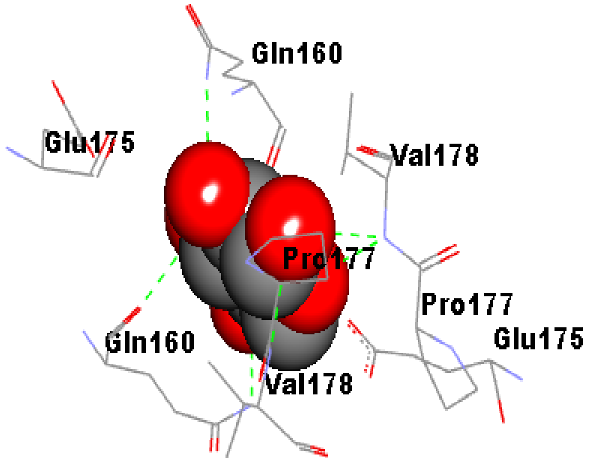

(f)

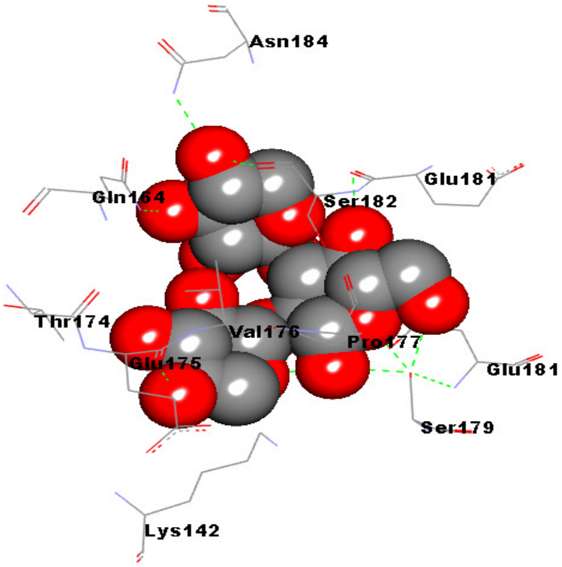

(g)

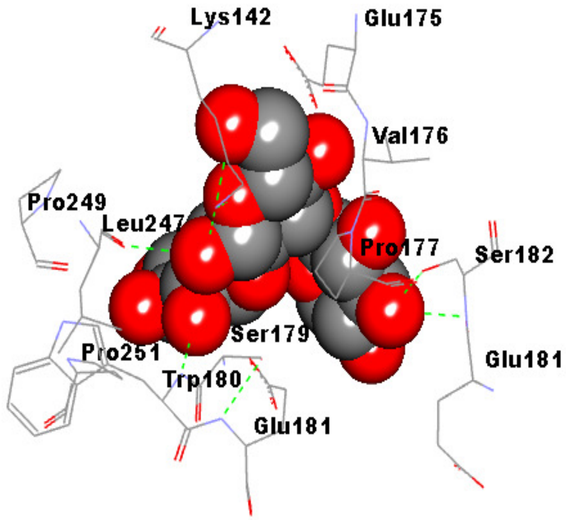

(h)

Best docking poses for binding of (a) solanidine, (b) solasodine, (c) alpha-solanine, (d) solasonine, e) solamargine, (f) degalactotigonin, (g) nigrumnin-I and (h) uttroside B with Gelsolin. The poses were generated using AutoDock v4.2.6.
