# Supplementary material for: Steroidal glycoalkaloids from Solanum nigrum target cytoskeletal proteins: an in silico analysis
Source: PeerJ. 2019 Jan 3;7:e6012. doi: 10.7717/peerj.6012 (PMC6321755; doi:10.7717/peerj.6012)
Supplement: Figure S9 — The poses were generated using AutoDock v4.2.6. [file peerj-07-6012-s009.pdf]

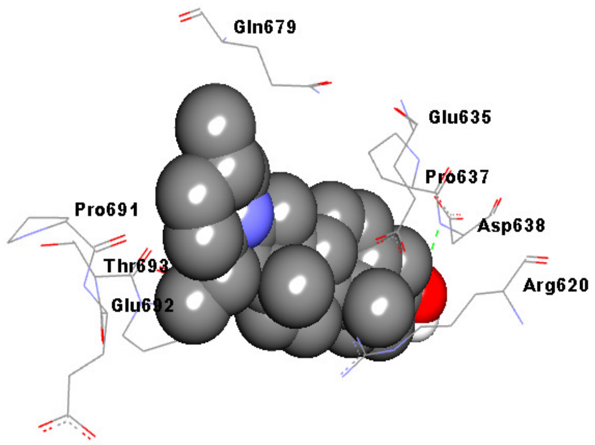
Gln679
Glu635
Pro637
Asp638
Arg620
Pro691
Thr693
Glu692

(a)

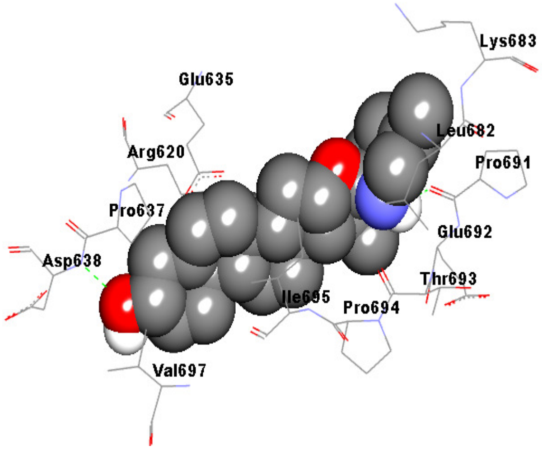
Lys683
Glu635
Leu682
Arg620
Pro691
Pro637
Glu692
Asp638
Thr693
Ile695
Pro694
Val697

(b)

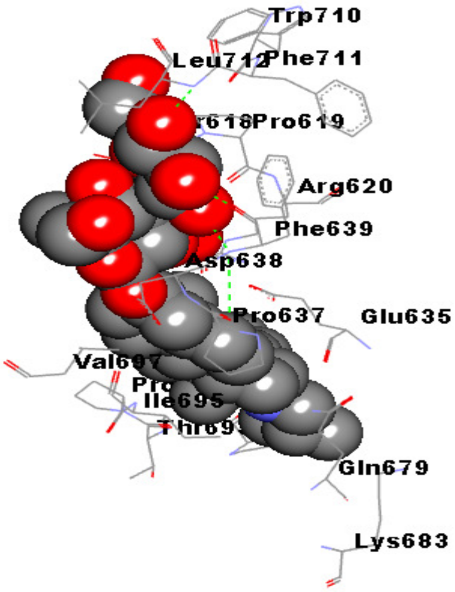
Trp710
Leu712
Phe711
Pro619
Arg620
Phe639
Asp638
Pro637
Glu635
Val697
Ile695
Gln679
Lys683

(c)

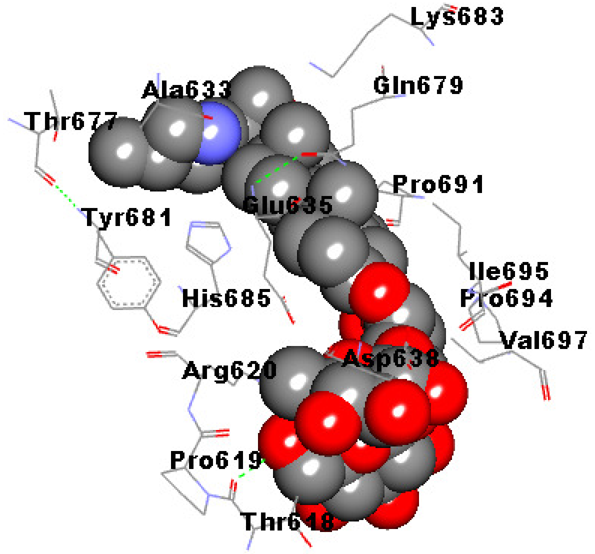
Lys683
Ala633
Gln679
Thr677
Pro691
Tyr681
Glu635
Ile695
Pro694
His685
Val697
Arg620
Asp638
Pro619
Thr618

(d)

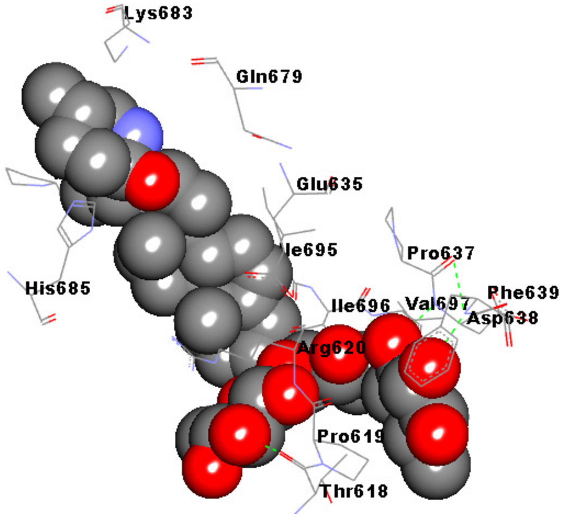

(e)

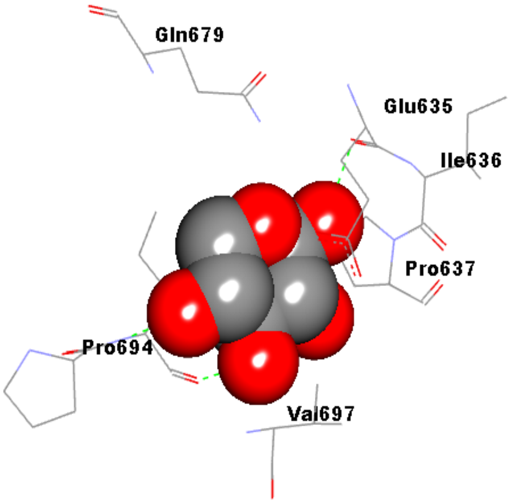

(f)

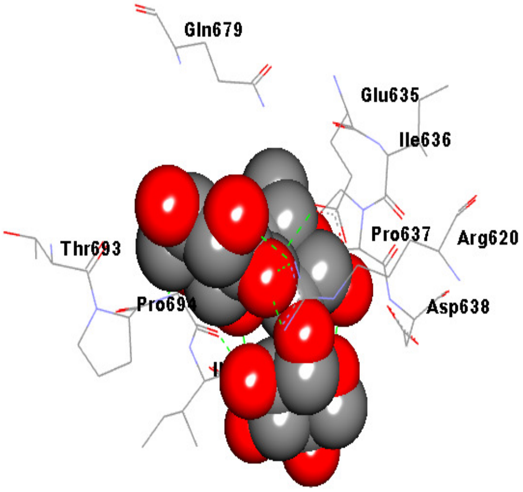

(g)

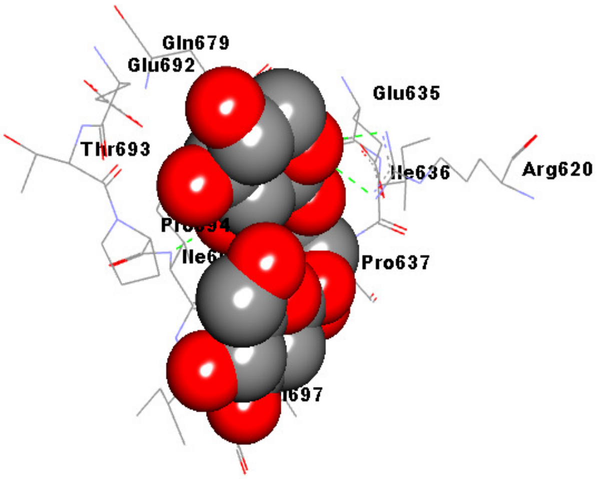

(h)

Best docking poses for binding of (a) solanidine, (b) solasodine, (c) alpha-solanine, (d) solasonine, e) solamargine, (f) degalactotigonin, (g) nigrumnin-I and (h) uttroside B with Villin. The poses were generated using AutoDock v4.2.6.
