# Supplementary material for: Steroidal glycoalkaloids from Solanum nigrum target cytoskeletal proteins: an in silico analysis
Source: PeerJ. 2019 Jan 3;7:e6012. doi: 10.7717/peerj.6012 (PMC6321755; doi:10.7717/peerj.6012)
Supplement: Figure S10 — The poses were generated using AutoDock v4.2.6. [file peerj-07-6012-s010.pdf]

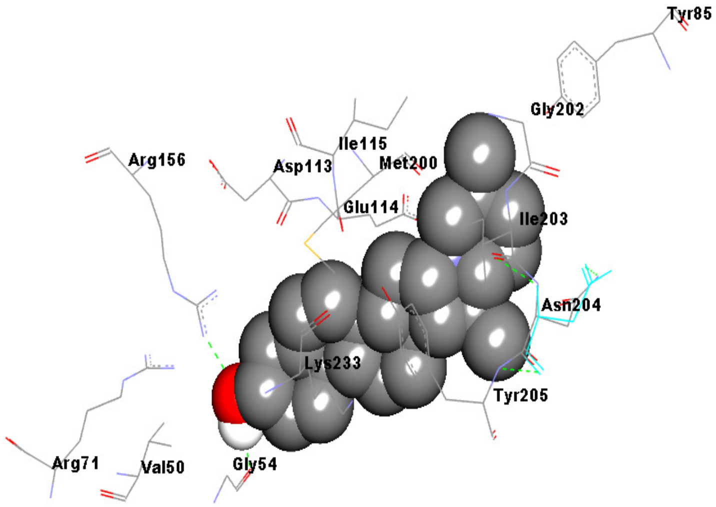
Tyr85
Gly202
Arg156
Ile115
Asp113
Met200
Glu114
Ile203
Asn204
Lys233
Tyr205
Arg71
Val50
Gly54

(a)

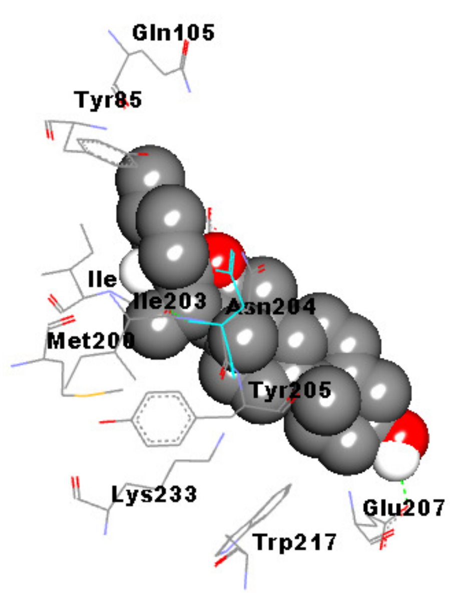
Gln105
Tyr85
Ile
Ile203
Asn204
Met200
Tyr205
Lys233
Glu207
Trp217

(b)

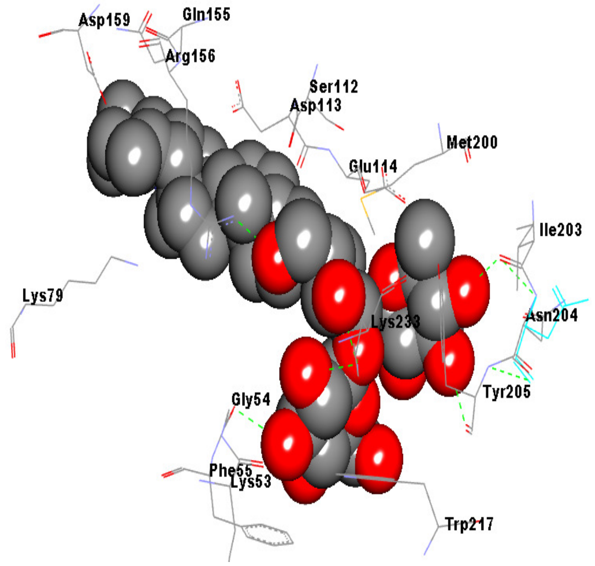
Asp159
Gln155
Arg156
Ser112
Asp113
Met200
Glu114
Ile203
Lys79
Lys233
Asn204
Tyr205
Gly54
Phe55
Lys53
Trp217

(c)

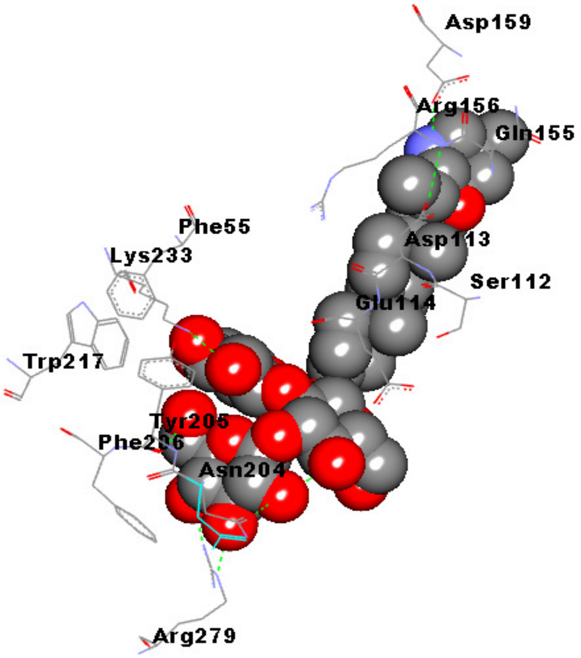
Asp159
Arg156
Gln155
Phe55
Asp113
Lys233
Ser112
Glu114
Trp217
Tyr205
Asn204
Arg279

(d)

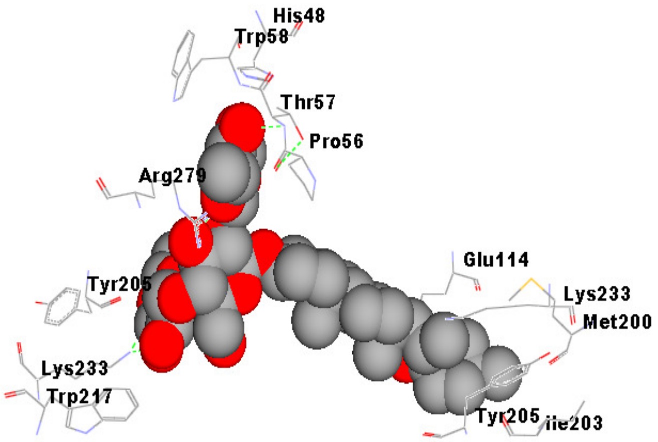


(e)

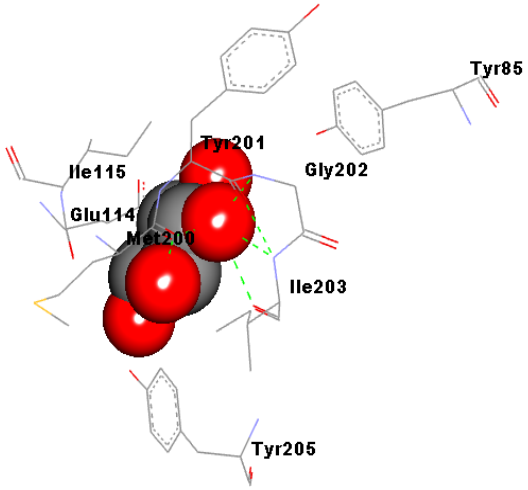


(f)

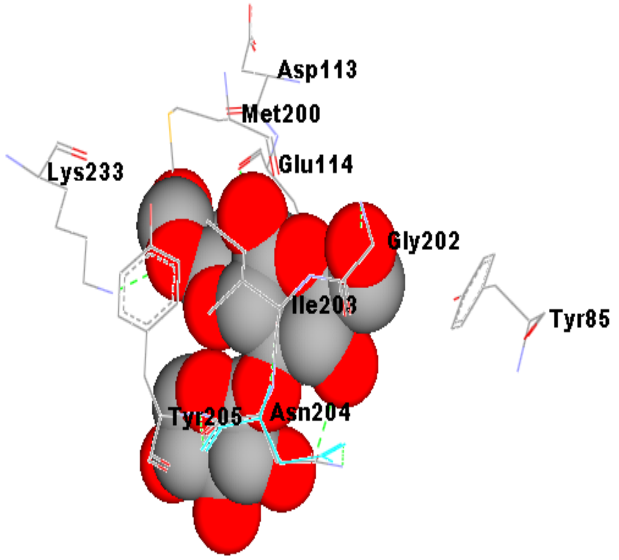


(g)

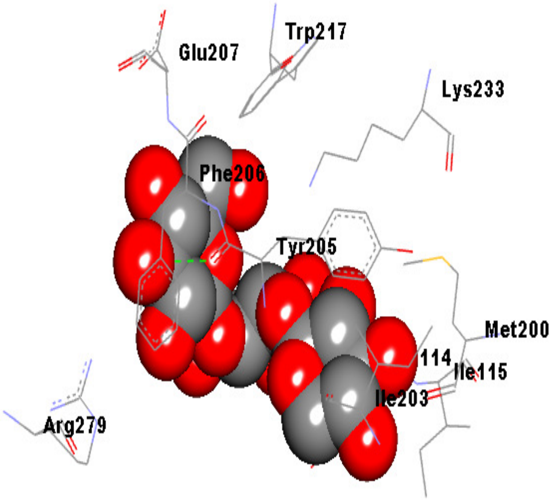


(h)

**Best docking poses for binding of (a) solanidine, (b) solasodine, (c) alpha-solanine, (d) solasonine, e) solamargine, (f) degalactotigonin, (g) nigrumnin-I and (h) uttroside B, with Ezrin. The poses were generated using AutoDock v4.2.6.**
