# Supplementary material for: Steroidal glycoalkaloids from Solanum nigrum target cytoskeletal proteins: an in silico analysis
Source: PeerJ. 2019 Jan 3;7:e6012. doi: 10.7717/peerj.6012 (PMC6321755; doi:10.7717/peerj.6012)
Supplement: Figure S11 — The pose was generated using AutoDock v4.2.6. [file peerj-07-6012-s011.pdf]

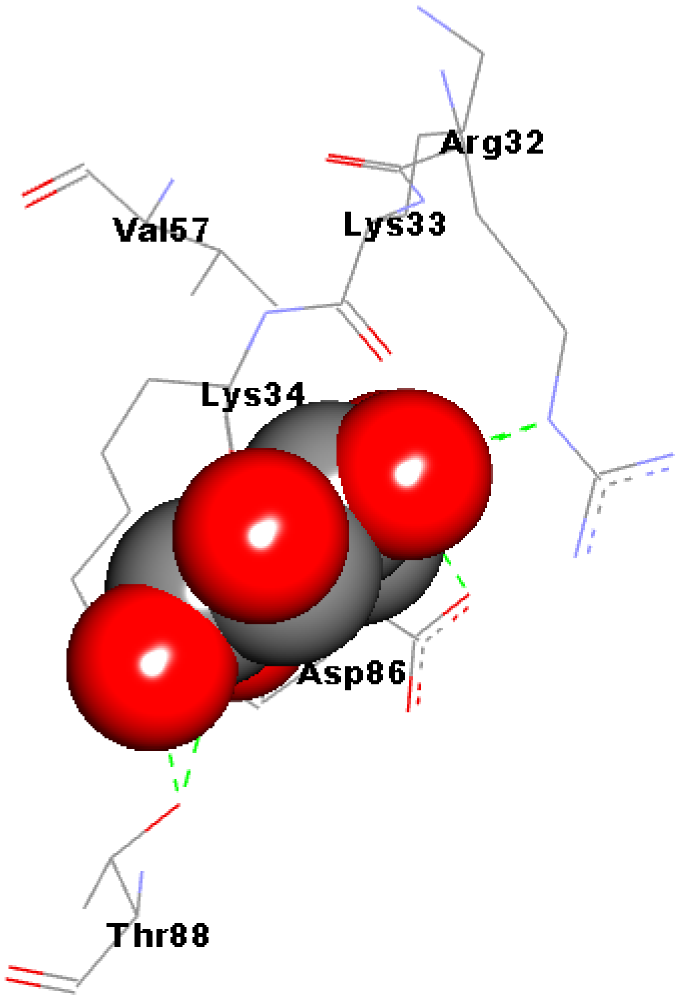

Best docking pose for binding of degalactotigonin with Cofilin-1. The pose was generated using AutoDock v4.2.6.
