# Supplementary material for: Steroidal glycoalkaloids from Solanum nigrum target cytoskeletal proteins: an in silico analysis
Source: PeerJ. 2019 Jan 3;7:e6012. doi: 10.7717/peerj.6012 (PMC6321755; doi:10.7717/peerj.6012)
Supplement: Table S2 [file peerj-07-6012-s023.docx]

| **All-Atom**  **Contacts** | Clashscore, all atoms: | 0 | | 100^th^ percentile* (N=1784, all resolutions) |
| --- | --- | --- | --- | --- |
|  | Clashscore is the number of serious steric overlaps (>0.4 Å) per 1000 atoms. | | | |
| **Protein Geometry** | Poor rotamers | 1 | 2.94% | Goal: <0.3% |
|  | Favored rotamers | 32 | 94.12% | Goal: >98% |
|  | Ramachandran outliers | 0 | 0.00% | Goal: <0.05% |
|  | Ramachandran favored | 34 | 100.00% | Goal: >98% |
|  | Molprobity score | 0.86 | | 100^th^ percentile* (N=27675, 0 Å - 99 Å) |
|  | Cβ deviations >0.25 Å | 0 | 0.00% | Goal: 0 |
|  | Bad bonds: | 0/297 | 0.00% | Goal: 0% |
|  | Bad angles: | 2/394 | 0.51% | Goal: <0.1% |
| **Peptide Omegas** | Cis Prolines: | 0/28 | 0.00% | Expected: ≤1 per chain, or ≤5% |

In the two column results, the left column gives the raw count, right column gives the percentage.

*100^th^ percentile is the best among structures of comparable resolution; 0^th^ percentile is the worst. For clashscore the comparative set of structures was selected in 2004, for Molprobity score in 2006

Molprobity score combines the clashscore, rotamer, and Ramachandran evaluations into a single score, normalized to be on the same scale as X-ray resolution
