# Supplementary material for: Steroidal glycoalkaloids from Solanum nigrum target cytoskeletal proteins: an in silico analysis
Source: PeerJ. 2019 Jan 3;7:e6012. doi: 10.7717/peerj.6012 (PMC6321755; doi:10.7717/peerj.6012)
Supplement: Data S1 [file peerj-07-6012-s024.docx]

https://www.rcsb.org/structure/1GK4

https://www.rcsb.org/structure/3FFN

https://www.rcsb.org/structure/3FG7

https://www.rcsb.org/structure/4RMA

https://www.rcsb.org/structure/4BAX
